# Enterotoxigenic *Escherichia coli* (ETEC) Infection Triggers Pyroptosis Through ER Stress Response-Mediated Mitochondrial Impairment and STING Activation in Intestinal Epithelial Cells

**DOI:** 10.3390/biology14121653

**Published:** 2025-11-23

**Authors:** Wenjie Yang, Xi Qiu, Jianan Guo, Yongxiang Wang, Jie Wang, Hongliang Chen, Di Zhang, Lei Zhang

**Affiliations:** 1 College of Veterinary Medicine, Jilin Agricultural University, Changchun 130118, China; ywj3512097072@163.com (W.Y.); 18084162935@163.com (X.Q.); 13086847475@163.com (J.G.); 17685931551@163.com (Y.W.); wj18455837439@163.com (J.W.); hongliang.chen@jlau.edu.cn (H.C.); 2 Engineering Research Center of Microecological Vaccines (Drugs) for Major Animal Diseases, Ministry of Education, Jilin Agricultural University, Changchun 130118, China

**Keywords:** ETEC, pyroptosis, ER stress, mitochondria, STING, intestinal epithelial cells (IECs)

## Abstract

Enterotoxigenic *Escherichia coli* (ETEC) is a major cause of diarrhea in pigs. Virulence factors, such as colonization factors and enterotoxins, bind to specific receptors on intestinal epithelial cells (IECs), impairing the integrity of the IEC barrier by inducing cell death. Pyroptosis is a newly discovered form of programmed cell death (PCD), which is widely involved in the pathogenesis of multiple infectious gastrointestinal diseases. However, it is still unclear whether pyroptosis contributes to the ETEC-mediated damage of IECs. This study demonstrated that ETEC infection activated NLRP3 inflammasome and triggered gasdermin D (GSDMD)-executed pyroptosis of mouse IECs in vitro and in vivo. Mechanistically, ETEC infection triggered endoplasmic reticulum (ER) stress response to increase the expression of thioredoxin-interacting protein (TXNIP) by upregulation of C/EBP homologous protein (CHOP), which subsequently activated NLRP3 inflammasome. Removal of ER stress by tauroursodeoxycholic acid (TUDCA) alleviated the pyroptosis of IECs that was caused by ETEC infection. In addition, the induced ER stress impaired mitochondria and led to mitochondrial reactive oxygen species (mtROS) overproduction and cytosolic release of mitochondrial DNA (mtDNA), which activated STING, another factor that contributed to ETEC-triggered pyroptosis. Chemical inhibition of STING attenuated ETEC-induced pyroptosis of IECs. Collectively, this study demonstrated that the activation of the STING/ER stress/mitochondrial impairment/NLRP3 inflammasome axis is a critical pathway in the ETEC infection-derived pyroptosis of IECs. Hence, targeting ER stress response may serve as a promising therapeutic strategy to prevent ETEC infection caused damage to IECs.

## Simple Summary

Infectious diarrhea disease has long been one of the leading causes of morbidity and mortality in the swine industry. Enterotoxigenic *Escherichia coli* (ETEC) is one of the major pathotypes of *E. coli* that confers the ability to cause diarrheal diseases. Prevention of *E. coli* infection is extremely important to maintain the growth performance and welfare of pigs. This study showed that ETEC activated the NLRP3 inflammasome and triggered pyroptosis in the intestinal epithelial cells (IECs) of mice. Activation of ER stress was found to be essential for ETEC-related pyroptosis by impairing mitochondria and activating STING signaling. Attenuation of ER stress with TUDCA ameliorated the inflammation and pyroptosis of IECs. These findings reveal that ER stress is a potential therapeutic target for ETEC-induced diarrheal disease.

## 1. Introduction

Enterotoxigenic *Escherichia coli* (ETEC) is the most common enteric pathogen and causes hundreds of millions of diarrheal illnesses annually in livestock worldwide. Acute watery diarrhea-associated rapid dehydration and prostration contribute to its high incidence of mortality [1]. To initiate infection, ETEC adheres to intestinal epithelial cells (IECs) to achieve colonization. Then, it secretes enterotoxins to damage the epithelial barrier function [1]. The impairment of intestinal integrity facilitates the penetration of toxins and pathogens, which exacerbates the intestinal damage by inducing severe inflammation [2]. Recent studies have revealed that ETEC infection promoted the release of inflammatory cytokines and triggered local and systemic immune response, which resulted damage of cells and tissues [3,4]. The cell death of IECs is a critical regulation of immune response and pathogenesis in inflammatory intestinal diseases [5,6].

Pyroptosis is a lytic form of programmed cell death (PCD), which is essential in defending against intracellular infections [7]. Canonically, pyroptosis is regulated by integration of inflammasome, whose assembly is activated in response to microbial infection [8]. The inflammasome is a multiprotein complex, consisting of a sensor protein, an adaptor protein, and caspase-1 [9]. NLRP3 is one of the most important inflammasome sensors, recognizing a broad spectrum of pathogen-associated molecular patterns (PAMPs) and toxins. The detection of a microbial signal promotes the assembly of pro-casapse-1 and its auto-cleaved activation, another defining feature of pyroptosis. Activated caspase-1 catalyzes the downstream cascades, including gasdermin D (GSDMD) cleavage and IL-18 and IL-1β maturation [8,10]. Previous studies have unveiled that the pyroptosis of IECs triggered severe inflammation in intestinal diseases [6,11,12]. Multiple pathogen bacteria have been observed to trigger pyroptosis [4,13,14]. It has been shown that ETEC infection activated NLRP3 inflammasome and triggered pyroptosis in murine intestinal tract [4].

Endoplasmic reticulum (ER) stress arises from the abnormal accumulation of misfolded or unfolded proteins due to inadequate protein-folding capacity [15,16]. This event subsequently provokes the activation of an unfolded protein response (UPR), whose hyperactivation results in cell death [15,16]. Emerging studies demonstrate that ER stress is a potent inducer of NLRP3 inflammasome and pyroptosis. Excessive calcium release from ER impaired mitochondrial function, leading to mitochondrial reactive oxygen species (mtROS) overproduction, which activated NLRP3 inflammasome [17]. Thioredoxin-interacting protein (TXNIP) is a critical signaling node that links ER stress and inflammatory cell death. PERK and IRE1 pathways of UPR activated TXNIP to provoke NLRP3 inflammasome and pyroptosis [18,19]. In addition, the PERK and IRE1 pathways also cooperated to activate CHOP-dependent inflammasome pyroptotic death in hepatocytes [20]. IECs are highly secretory cells, which are susceptible to ER stress-dependent inflammation. For example, the deletion of XBP1 in IECs, a key transcriptional factor of UPR, promoted the development of intestinal inflammation spontaneously [21].

The relationship between ETEC infection and pyroptosis, and its potential mechanism, remains unclear. Whether ER stress contributes to ETEC infection-induced pyroptosis is also unknown. In this study, we explored the role of ER stress in the ETEC-induced pyroptosis of IECs. Our results demonstrate that ETEC infections led to NLRP3 inflammasome-dependent, GSDMD-executed pyroptosis in IECs by activating ER stress response. ER stress impaired mitochondria, which promoted the production of ROS. Moreover, the stimulator of interferon gene (STING) was activated, emerging as an upstream modulator of ETEC-mediated ER stress response, which contributed to ETEC-induced pyroptosis of IECs.

## 2. Materials and Methods

### 2.1. Cells and Bacteria

The mouse intestinal epithelial cell line, Mode-k, was cultured in Dulbecco’s Modified Eagle Medium (DMEM) (Gibco, Grand Island, NY, USA) supplemented with 10% fetal bovine serum (Gibco, Grand Island, NY, USA) and antibiotics (100 units/mL penicillin and 100 mg/L streptomycin) (Invitrogen, Carlsbad, CA, USA) at 37 °C in a humidified incubator containing 5% CO_2_. The ETEC Standard Strain C83902 (O8: K887: K88ac) was a generous gift from Guoqiang Zhu from the College of Veterinary Medicine of Yangzhou University. ETEC were cultured in Luria–Bertani (LB) media at 37 °C.

### 2.2. Animal Experiments

Healthy C57BL/6 male mice (6–8 weeks old) were obtained from Huafukang. All mice were housed in plastic cages with chow diet and water ad libitum under standard conditions. All animals were adapted to the environment for 1 week before the treatment. The mice were randomly divided into two groups. The control group (4 mice) received 200 μL PBS via intragastric gavage, and the ETEC group (4 mice) received 200 μL of 1 × 10^10^ CFU/mL ETEC via intragastric gavage. The treatments were conducted three times a day and kept for 5 days.

To investigate whether ER stress contributes to ETEC-induced pyroptosis in vivo, C57BL/6 mice were randomly divided into four groups. The control group (4 mice) was received 200 μL of PBS via intragastric gavage. The ETEC group (4 mice) received 200 μL of 1 × 10^10^ CFU/mL ETEC via intragastric gavage. The TUDCA group (4 mice) received 150 μg/g TUDCA (HY-19696, MCE, Shanghai, China) via intragastric gavage. The ETEC + TUDCA group (4 mice) received 200 μL of 1 × 10^10^ CFU/mL ETEC with 150 μg/g TUDCA via intragastric gavage. The treatments were conducted 3 times a day and kept for 5 days.

After the treatments, the animals were euthanized under anesthesia. A portion of the intestinal tissue was rapidly frozen in liquid nitrogen and stored at −80 °C for further analysis, while the remaining tissue was fixed in a solution containing 4% paraformaldehyde for histological examination.

All of the animal procedures in this study were reviewed and authorized by the Animal Protection and Ethics Committee of Jilin Agricultural University (JLAU20220610001) and were conducted in compliance with protocols approved by local ethical government authorities.

### 2.3. In Vitro Assay for Cell Viability

Cell death was measured by CCK-8 assay (HY-K0301, MCE, Shanghai, China). Mode-k cells (1 × 10^5^ cells/well) were seeded in 96-well plates in 100 μL DMEM medium. After the respective treatments, 10 μL CCK-8 solution was added to each well and incubated at 37 °C for 4 h. The optical density (OD) was measured at 450 nm by a microplate reader (Biotek HTX, Beijing, China). Cell viability was evaluated as (OD value of the stimulated cell-OD value of unstimulated cells)/(OD value of the unstimulated cell-OD value of blank) × 100%. The cell death rate was adjusted by cell viability.

### 2.4. Real-Time Quantitative PCR (qPCR) Analysis

Total RNA was extracted from mode-k cells and intestinal tissues using TRIzol^®^ reagent (Takara, Beijing, China) following the protocol. The quality and quantity of the RNA was measured by NanoDrop 1000 (Thermo Fisher, Shanghai, China). cDNA synthesis was performed with the First Strand cDNA Synthesis Kit (D7168L Beyotime, Shanghai, China). Quantitative PCR reactions utilized SYBR Green MasterMix (Beyotime, Shanghai, China) on an Applied Biosystems 7500 Real-Time PCR System (Thermo Fisher, Shanghai, China). The results are displayed as relative expression values normalized to that of GAPDH. Relative gene quantification was calculated using the 2^−ΔΔCt^ method. The specific primer sequences are provided in Table 1.

### 2.5. Western Blot

After treatment, cells and tissues were harvested and lysed with a BBproExtro protein extraction kit (BB-3121, Bestbio, Shanghai, China). After centrifugation (16,000× *g* for 20 min at 4 °C), the supernatants were collected and quantitated by BCA protein assay (P0010, Beyotime, Shanghai, China) according to the manufacturer’s instructions. Equal amounts of protein (20 μg) with lammli sample buffer were loaded to SDS-PAGE gels for electrophoresis and transferred to PVDF membrane. The membrane was then washed with TBST and blocked with 2% bovine serum albumin (BSA) for 1 h. After that, the membrane was incubated with the primary antibodies targeting BIP (AF5366, Affinity Bioscience, Zhenjiang, China), CHOP (AF6277, Affinity Bioscience, Zhenjiang, China), PERK (A18196, Abclonal, Wuhan, China), NLRP3 (A5620, Abclonal, Wuhan, China), Caspase-1 (AF5418, Affinity Bioscience, Zhenjiang, China), GSDMD (AF4012, Affinity Bioscience, Zhenjiang, China), TXNIP (AF8277, Beyotime, Shanghai, China), STING (YP1684, Abclonal, Wuhan, China), Phospho-STING (AP1369, Abclonal, Wuhan, China), cGAS (A8335, Abclonal, Wuhan, China) and GAPDH (AF7021, Affinity Bioscience, Zhenjiang, China) ovenight at 4 °C. After TBST wash, the membrane was incubated with a secondary antibody at room temperature for 1 h. Protein signals were detected using an enhanced chemiluminescence (ECL) substrate (WBKLS0100, Millipore, Billerica, MA, USA) and an imaging system (Invitrogen iBright FL1500, Thermo Fisher Scientific, Shanghai, China). The experiment was performed in triplicate.

### 2.6. Measurement of Cytokines and Cytochrome C by ELISA

The IL-1β and IL-18 level was measured via ELISA according to instructions provided with the ELISA kits (YX-E20533 and YX-E11385, Sinobestbio, Shanghai, China). The sensitivity of the ELISA kit is 1 pg/mL. For in vitro test, mode-k cells (5 × 10^5^) were seeded in a 6-well plate. After respective treatments, the cell supernatant was collected by centrifugation (1500 rpm, 15 min, 4 °C) after the treatment. The IL-1β and IL-18 in the cell supernatant of each group was measured. For the in vivo test, levels of IL-1β and IL-18 from mouse serum were measured after their respective treatments. Absorbance was measured at 450 nm using a microplate reader (Biotek HTX, Beijing, China). Lipopolysaccharides (LPS) (HY-D1056, MCE, Shanghai, China) and ATP (HY-B2176, MCE, Shanghai, China) cotreatment was used as a positive control to validate the ELISA sensitivity of IL-1β and IL-18.

The cytosolic cytochrome c levels were quantified following subcellular fractionation. The cytosolic fraction was prepared using the cell mitochondria isolation kit (C3601, Beyotime, Shanghai, China), according to the manufacturer’s guidelines. Briefly, cells were collected and centrifuged at 900× *g* for 5 min. The supernatant was discarded. The pellet was homogenized with lysis buffer for 10 min on ice. The homogenate was centrifuged at 700× *g* for 10 min at 4 °C. We collected the supernatant and discarded the pellet. Then, we transferred the supernatant and centrifuged at 10,000× *g* for 30 min at 4 °C to isolate cytosolic fraction. Quantification of cytochrome c in the cytosolic fraction was performed using the mouse Cytochrome c ELISA Kit (EM0977, FineTest, Wuhan, China). Absorbance was measured at 450 nm using a microplate reader, and concentrations were interpolated from the standard curve generated in parallel.

### 2.7. ER-Tracker Analysis

Mode-k cells were treated, collected, and resuspended (1 × 10^6^ cells/mL) in complete DMEM medium. ER-Tracker (500 nM) (C1041M, Beyotime, Shanghai, China) was added to the resuspended cells and incubated for 30 min at 37 °C. Counterstaining was performed with DAPI. Fluorescence images of ER were captured using a confocal microscope (Leica, Neunkhausen, Germany), and fluorescence intensity was quantified by ImageJ (version 1.53). One hundred cells per sample were counted.

### 2.8. Mitochondrial Membrane Potential Damage Analysis

JC-1 staining (40705ES03, Yeasen Biotechnology, Shanghai, China) was used to measure the mitochondrial membrane potential according to the manufacture’s instruction. Briefly, mode-k cells were collected after the respective treatments. Then the cells were stained with the JC-1 and DAPI for 30 min at 37 °C and detected with a confocal microscope (Leica, Neunkhausen, Germany). Red emission of the dye represented the aggregated form of JC-1 (excitation = 585 nm and emission = 590 nm), and green emission represented the monomeric form of JC-1 (excitation = 514 nm and emission = 529 nm), which indicated mitochondrial membrane depolarization. One hundred cells per sample were counted.

### 2.9. Measurement of Mitochondrial Superoxide

Mitochondrial superoxide was detected with the MitoSox Red Mitochondrial Superoxide indicator dye (40778ES50, Yeasen Biotechnology, Shanghai, China). Mode-k cells were cultured in 6-well plates overnight and treated as described for 24 h. After the treatment, cells were gently collected and stained with MitoSox and DAPI for 30 min at 37 °C. The fluorescent images of cells were captured using a confocal microscope (Leica, Neunkhausen, Germany). The relative fluorescent intensity was analyzed with the ImageJ software (version 1.53). One hundred cells per sample were counted.

### 2.10. Detection of Cytosolic mtDNA by qPCR

Cells were collected and incubated with NP-40 lysis buffer for 15 min at 4 °C. Then, the mixture was centrifuged at 13,500× *g* for 15 min at 4 °C. The supernatant was transferred to a new EP tube as a cytosolic fraction, and the remaining pellet represented the organelle fraction. DNA was isolated using phenol:chloroform. Different fractions were resuspended in TRIzol reagent and incubated for 5 min at room temperature. Chloroform (1:5 Chlor:TRIzol) was added and fractions were incubated for another 3 min. Samples were centrifuged for 15 min at 12,000× *g* at 4 °C and the RNA-containing fraction was discarded. Pure ethanol was added, mixed and incubated for 3 min. A DNA pellet was collected by centrifuging for 5 min at 2000× *g* at 4 °C. The pellet was resuspended in 75% ethanol, incubated for 15 min, and centrifuged for 5 min at 2000× *g* at 4 °C. The DNA was air-dried and resuspended in NE buffer. The DNA was qualified and quantified by NanoDrop 1000 (Thermo Fisher, Shanghai, China). We prepared the SYBR qPCR master mix to measure the mtDNA (ND-1) and internal control (18S). The primers are listed below in Table 2. The mtDNA abundance was relative to nuclear DNA, which was calculated using the delta delta Cq (ΔΔCq) method.

### 2.11. Histological Evaluation

To evaluate intestinal morphology, intestinal tissue samples were fixed in 4% paraformaldehyde, dehydrated, paraffin embedded, sectioned into 5 μm slices, and stained with haematoxylin and eosin (H&E). The histological and morphological changes in the intestinal tissue were observed under an upright microscope (Leica, Neunkhausen, Germany).

### 2.12. Statistical Analysis

All experiments were conducted at least three times, and the data are presented as the mean ± SD. Statistical significance among different treatments were determined by one-way ANOVA followed by Tukey’s post hoc test. A *p* or adjusted *p* value < 0.05 is considered statistically significant.

## 3. Results

### 3.1. ETEC Activates NLRP3 Inflammasome and Induces Pyroptosis in Murine IECs

**Figure 1:**
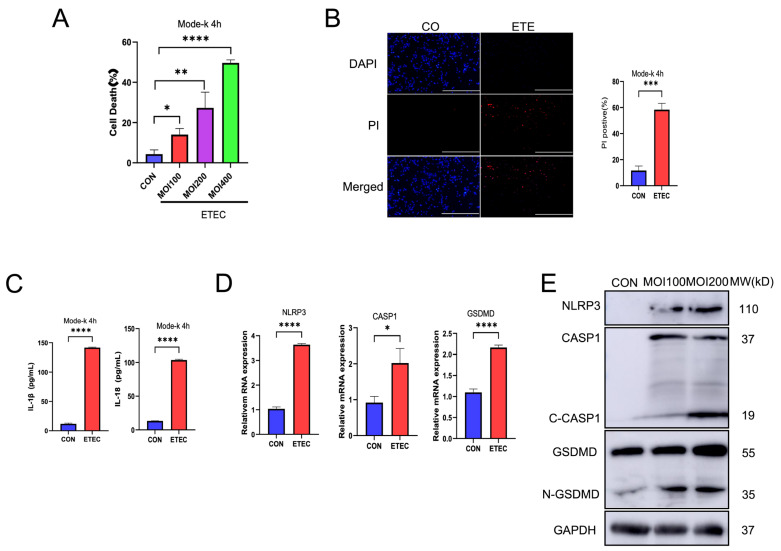
ETEC infection triggers pyroptosis in mode-k cells. (**A**) An increased dose of ETEC (MOI 100, 200 and 400) was used to treat mode-k cells for 4 h (n = 3). Cell death was analyzed by CCK-8 assay. (**B**) Cell death was measured by quantifying the percentage of propidium iodide (PI)-positive cells after treatment with MOI 200 ETEC for 4 h (n = 3). (**C**) Supernatant cytokine levels were quantified for IL-1β and IL-18 in mode-k cells after treatment of MOI 200 ETEC for 4 h (n = 3). (**D**) Relative expressions of NLRP3, CASP1, and GSDMD mRNA (normalized to GAPDH mRNA) were measured in mode-k cells after treatment of MOI 200 ETEC for 4 h (n = 3). (**E**) The expressions of NLRP3, active CASP1 and N-GSDMD were evaluated in mode-k cells after treatment of MOI 200 ETEC for 4 h (n = 3). Scale bar = 50 μm. Data are presented as mean ± SD. Differences were assessed by one-way ANOVA followed by Tukey’s tests. * *p* < 0.05, ** *p* < 0.01, *** *p* < 0.001, and **** *p* < 0.0001.

To investigate whether ETEC induces pyroptosis in IECs, we infected mode-k cells, a murine IEC cell line with ETEC at increased multiplicities of infection (MOIs) of 100, 200 and 400 for 4 h. ETEC infection led to a significant increase of cell death in an MOI-dependent manner in mode-k cells (Figure 1A). After analysis, we chose to use MOI 200 in the following experiment. MOI 200 induced accumulation of intracellular propidium iodide (PI) staining (Figure 1B). The following ELISA analysis demonstrated that MOI 200 ETEC infection induced mature IL-1β and IL-18 release from mode-k cells (Figure 1C), which indicated the activation of inflammatory cell death. Here, we also stimulated mode-k cells with cotreatment of LPS and ATP as a positive control to validate the ELISA sensitivity (Supplementary Figure S3). To further characterize the mode of inflammatory cell death that triggered by ETEC infection, we analyzed key molecules associated with NLRP3 inflammasome–GSDMD-dependent pyroptosis. MOI 200 ETEC infection significantly increased the mRNA levels of NLRP3, Caspase-1 (CASP1) and GSDMD (Figure 1D). Western blot (WB) analysis revealed that NLRP3 was significantly upregulated (Figure 1E) concomitantly with the active cleavage form of Caspase-1 (C-CASP1) and N-terminal GSDMD (N-GSDMD) (Figure 1E) after ETEC infection. Meanwhile, activation of pyroptosis was observed in a dose-dependent manner in mode-k cells infected with serial MOIs of ETEC (Figure 1E). Together, these results suggest that ETEC infection activates the NLRP3 inflammasome–GSDMD pathway to trigger pyroptosis in mode-k cells.

### 3.2. ETEC Induces ER Stress in IECs

Because ETEC bacteria was too toxic to Mode-k cells in very short time, we applied ETEC lysate to treat the cells to explore the signaling pathways that were modulated by ETEC. ETEC was ultrasonically lysed to generate ETEC lysate when the bacteria were grown to OD_600_ = 1.0 (10^9^ bacteria/mL). ETEC lysate was applied to treat the cells for 24 h in a ratio of 50 µL ETEC lysate for 50,000 cells. Our results demonstrated that ETEC lysate induced pyroptosis in Mode-k cells after 24 h treatment (Supplementary Figure S1A–C).

**Figure 2:**
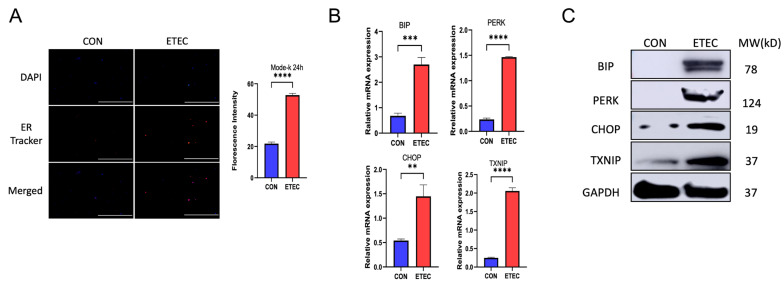
ETEC induces ER stress response in Mode-k cells. ETEC lysate was used to treat Mode-k cells for 24 h. (**A**) Fluorescent ER tracker was used to stain the Mode-k cells. Fluorescent images were captured, and fluorescence intensities were analyzed by ImageJ (n = 3). (**B**) Relative expressions of BIP, PERK, CHOP and TXNIP mRNA (normalized to GAPDH mRNA) were measured in Mode-k cells (n = 3). (**C**) The expressions of BIP, PERK, CHOP, and TXNIP were evaluated in Mode-k (n = 3). Scale bar = 50 μm. Data were presented as Mean ± SD. Differences were assessed by one-way ANOVA followed by Tukey’s tests. ** *p* < 0.01, *** *p* < 0.001, and **** *p* < 0.0001.

ER stress response is a major contributor to inflammatory diseases. To determine whether ETEC infection induced ER stress in Mode-k cells, we first stained the Mode-k cells with fluorescent ER probe and quantified the fluorescent intensity after treated with ETEC lysate for 24 h. Enhanced fluorescence is an indicative of ER stress [22]. The results showed that ETEC lysate significantly increased the fluorescence intensity of ER tracker (Figure 2A). Meanwhile, ETEC also induced the activation of UPR, as indicative by an elevation in transcript and protein levels of BIP, PERK and CHOP (Figure 2B,C). TXNIP is a critical link node between ER stress and NLRP3 inflammasome. Three signal transducers of UPR, ATF6, IRE1 and PERK, promote the CHOP-dependent activation of TXNIP, which subsequently interacts with NLRP3, stimulating NLRP3 inflammasome formation and pyroptosis [23]. Our results indicated that ETEC lysate treatment upregulated mRNA transcript and protein expression of TXNIP in Mode-k cells (Figure 2B,C), which was in accordance with the upregulation of CHOP. Taken together, our results indicated that ETEC induced ER stress and activated TXNIP in Mode-k cells.

### 3.3. ETEC Induces Intestinal Pyroptosis and ER Stress In Vivo

**Figure 3:**
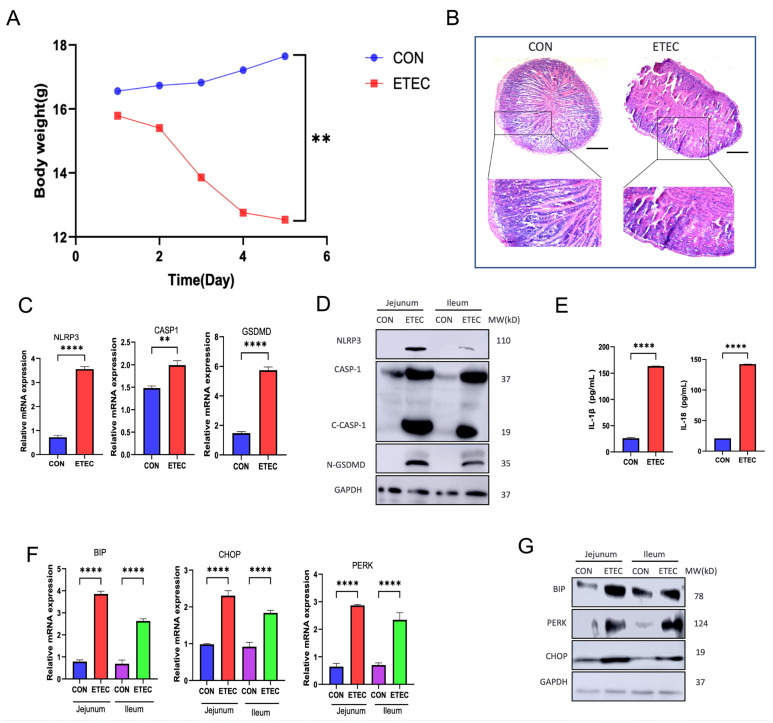
ETEC induces intestinal pyroptosis and ER stress response in vivo. Streptomycin-pretreated C57BL/6mice were orally infected with 200 μL PBS (CON n = 4) or 1 × 10^10^ CFU/mL ETEC (ETEC n = 4) three times a day for 5 days. (**A**) Body weight change. (**B**) Histopathological analysis of the intestine. (**C**) Relative expressions of NLRP3, CASP1, and GSDMD mRNA (normalized to GAPDH mRNA) were measured in intestinal tissues (n = 3). (**D**) The expressions of NLRP3, active CASP1 and N-GSDMD were evaluated in jejunum and ileum (n = 3). (**E**) Serum cytokine levels were quantified for IL-1β and IL-18 (n = 3). (**F**) Relative expressions of BIP, CHOP and PERK mRNA (normalized to GAPDH mRNA) were measured in jejunum and ileum (n = 3). (**G**) The expressions of BIP, PERK, and CHOP were evaluated in jejunum and ileum (n = 3). Scale bar = 250 μm. Data were presented as Mean ± SD. Differences were assessed by one-way ANOVA followed by Tukey’s tests. ** *p* < 0.01, and **** *p* < 0.0001.

Next, we investigated whether ETEC infection triggered intestinal pyroptosis and induced ER stress response in vivo. Streptomycin pretreated mice were orally infected with sterile PBS (CON) or ETEC for 5 days. We monitored the body weight change during the course pf infection. A gradual decrease of body weight in ETEC infected mice was observed comparing to control group as the infection processed (Figure 3A). After sacrificing the animals, we observed that ETEC caused bleeding in the collected jejunum and ileum tissues, and the stools of the infected mice were loose and soft (Supplementary Figure S2). The following hematoxylin and eosin (H&E) staining analysis demonstrated that ETEC induced severe villus atrophy and loss of IECs in the mucosal tissue, in parallel with infiltration of inflammatory cells into the lamina propria and submucosa (Figure 3B). The physiological and morphological results indicated that ETEC infection impaired intestinal integrity by inducing inflammation.

To explore whether ETEC-triggered pyroptosis of IECs involved in the intestinal damage in vivo, we collected intestinal tissues and analyzed the pyroptotic markers. ETEC challenge significantly upregulated mRNA transcripts and protein levels of NLRP3, Caspase-1 and GSDMD (Figure 3C,D). More importantly, ETEC activated Caspase-1 by showing cleaved-Caspase-1 and its substrate, the executer of pyroptosis, N-GSDMD was also increased in jejunum and ileum (Figure 3D). Meanwhile, the expression of Caspase-1-dependent inflammatory cytokines, IL-1β and IL-18, were also increased in the serum of ETEC infected mice (Figure 3E). These results indicated that ETEC stimulated NLRP3/Caspase-1/GSDMD signaling axis to execute pyroptosis in IECs of infected mice.

In addition, we examined the intestinal status of ER stress in ETEC challenged mice. ETEC infection promisingly activated UPR by showing a significant upregulation of mRNA transcripts and protein levels of UPR signaling members, BIP. PERK and CHOP (Figure 3F,G). The results suggested that ETEC induced ER stress in IECs of infected mice. Collectively, our results indicated ETEC induced pyroptosis and ER stress response in IECs in vivo.

### 3.4. TUDCA Protects the IECs from ETEC-Induced Pyroptosis by Alleviating ER Stress

**Figure 4:**
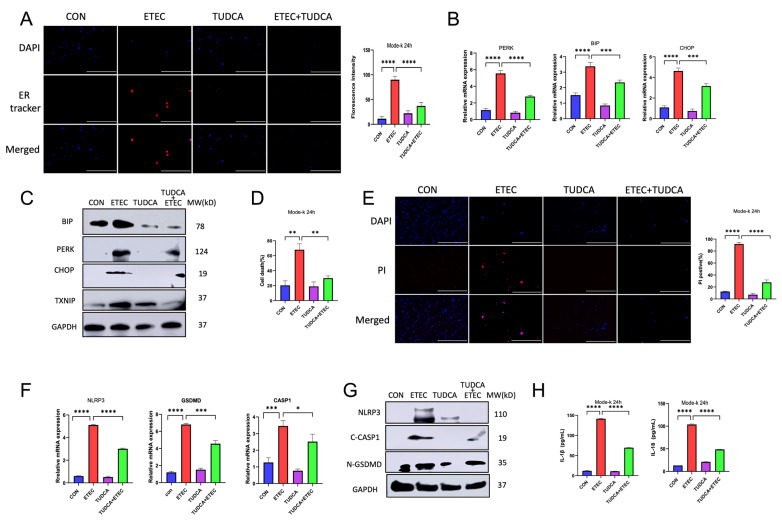
TUDCA inhibited ETEC induced pyroptosis by attenuating ER stress in vitro. ETEC lysate was used to treat Mode-k cells alone or in combination of TUDCA (800 μM) for 24 h. (**A**) Fluorescent ER tracker was used to stain the Mode-k cells. Fluorescent images were captured, and fluorescence intensities were analyzed by ImageJ (n = 3). (**B**) Relative expressions of PERK, BIP and CHOP mRNA (normalized to GAPDH mRNA) were measured (n = 3). (**C**) The expressions of BIP, PERK, CHOP, and TXNIP were evaluated (n = 3). (**D**) Cell death was analyzed by CCK-8 assay (n = 3). (**E**) Cell death was measured by quantifying the percentage of propidium iodide (PI) positive cells (n = 3). (**F**) Relative expressions of NLRP3, CASP1, and GSDMD mRNA (normalized to GAPDH mRNA) were measured (n = 3). (**G**) The expressions of NLRP3, active CASP1 and N-GSDMD were evaluated (n = 3). (**H**) Supernatant cytokine levels were quantified for IL-1β and IL-18 (n = 3). Scale bar = 50 μm. Data were presented as Mean± SD. Differences were assessed by one-way ANOVA followed by Tukey’s tests. * *p* < 0.05, ** *p* < 0.01, *** *p* < 0.001, and **** *p* < 0.0001.

Our previous results demonstrated the co-existence of pyroptosis and ER stress response in ETEC infected IECs both in vitro and in vivo. Considering the critical role of ER stress response in inflammation and pyroptosis, we next explored whether ER stress response contributed to ETEC-induced pyroptosis in IECs. Tauroursodeoxycholic acid (TUDCA) is an ER stress inhibitor, working as a molecular chaperone to promote protein folding [24]. We first investigated the in vitro effects of TUDCA on the pyroptosis that was induced by ETEC in Mode-k cells. TUDCA dramatically attenuated ETEC lysate induced ER stress response in Mode-k cells. As shown in Figure 4A, TUDCA decreased the fluorescence intensity of ER tracker that was upregulated by ETEC lysate in Mode-k cells. The ETEC lysate-induced upregulation of mRNA transcripts and protein expression of UPR members, PERK, BIP and CHOP were also downregulated by TUDCA, as well as TXNIP (Figure 4B,C). These results suggested that TUDCA efficiently alleviated ETEC-induced ER stress response. Meanwhile, we also found that TUDCA significantly attenuated the cell death that induced by ETEC lysate (Figure 4D,E). As expected, TUDCA attenuated ETEC lysate-caused inflammation and pyroptosis by showing a downregulation of the mRNA transcripts of NLRP3, Caspase-1 and GSDMD (Figure 4F), protein expression of NLRP3 (Figure 4G), and active executers of pyroptosis, cleaved Caspase-1 and N-GSDMD (Figure 4G). TUDCA also decreased ETEC-resulted upregulation of cytokines, IL-1β and IL-18 (Figure 4H).

**Figure 5:**
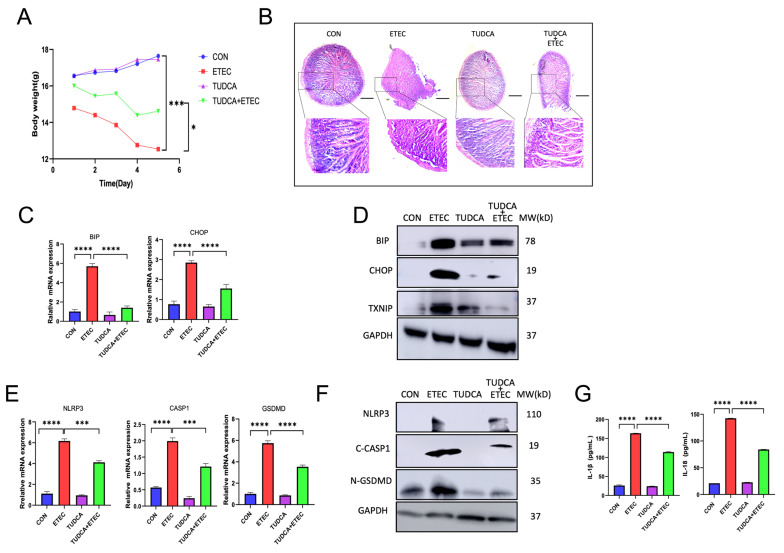
TUDCA inhibited ETEC induced pyroptosis by attenuating ER stress in vitro. Streptomycin-pretreated C57BL/6mice were orally infected with 200 μL PBS (CON n = 4) or 1 × 10^10^ CFU/mL ETEC (ETEC n = 4) three times a day for 5 days. Intragastric gavage of TUDCA 150 μg/g was received three times a day alone (TUDCA) or together with ETEC (TUDCA+ETEC) for 5 days. (**A**) Body weight change. (**B**) Histopathological analysis of the intestine. (**C**) Relative expressions of BIP and CHOP mRNA (normalized to GAPDH mRNA) were measured in intestinal tissues (n = 3). (**D**) The expressions of BIP and CHOP were evaluated in intestine (n = 3). (**E**) Relative expressions of NLRP3, CASP1 and GSDMD mRNA (normalized to GAPDH mRNA) were measured in intestine (n = 3). (**F**) The expressions of NLRP3, C-CASP1, and N-GSDMD were evaluated in intestine (n = 3). (**G**) Serum cytokine levels were quantified for IL-1β and IL-18 (n = 3). Scale bar = 250 μm. Data were presented as Mean ± SD. Differences were assessed by one-way ANOVA followed by Tukey’s tests. * *p* < 0.05, *** *p* < 0.001 and **** *p* < 0.0001.

Next, we explored the protective effects of TUDCA in vivo. Five days of TUDCA treatment partially restored the body weight of the mice that challenged with ETEC (Figure 5A). Intestinal histological analysis revealed that TUDCA attenuated the ETEC induced intestinal damage and infiltration of inflammatory cells (Figure 5B). Consistent with the in vitro results, TUDCA efficiently reduced the ETEC-induced ER stress response in vivo by demonstrating a downregulation of the key biochemical markers of UPR, BIP and CHOP, at transcriptional and protein levels (Figure 5C,D). TUDCA also downregulated the protein level of TXNIP in intestine. Meanwhile, TUDCA markedly decreased the mRNA transcript and protein expression of NLRP3, and the cleaved-Caspase-1 and N-GSDMD upon ETEC challenge (Figure 5E,F). The serum levels of IL-1β and IL-18 were also decreased after TUDCA co-treated with ETEC (Figure 5G). Collectively, our results indicated that ER stress response contributed to ETEC-induced pyroptosis in IECs.

### 3.5. ETEC Impairs Mitochondria in IECs

**Figure 6:**
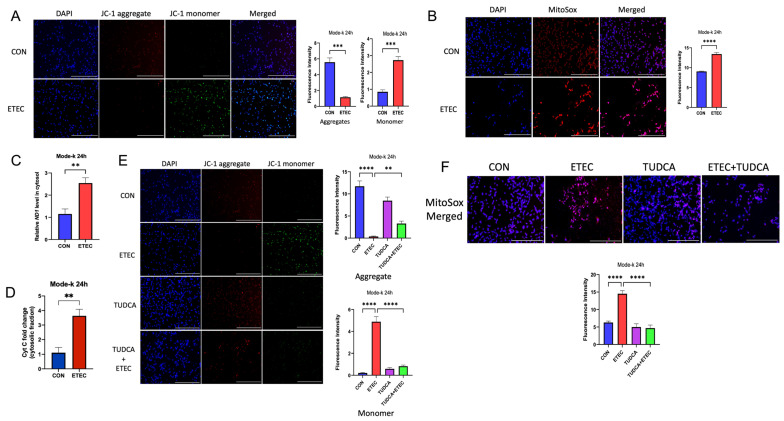
ETEC impairs mitochondrial integrity and promotes mitochondrial ROS production. ETEC lysate was used to treat Mode-k cells for 24 h. (**A**) JC-1 was used to measure the mitochondrial membrane potential (n = 3). (**B**) MitoSox was used to evaluate the production of mitochondrial ROS (n = 3). Fluorescent images were captured, and fluorescence intensities were analyzed by ImageJ. (**C**) qPCR was used to detect mtDNA levels (ND1) in the cytosol relative to nuclear DNA levels (18S) (n = 3). (**D**) The relative levels of cytosolic Cyt c expressed as the fold change compared with the proliferating control (n = 3). ETEC lysate (MOI 200) was used to treat Mode-k cells alone or in combination of TUDCA (800 μM) for 24 h. (**E**) JC-1 was used to measure the mitochondrial membrane potential (n = 3). (**F**) MitoSox was used to evaluate the production of mitochondrial ROS (n = 3). Fluorescent images were captured, and fluorescence intensities were analyzed by ImageJ. Scale bar = 50 μm. Data were presented as Mean ± SD. Differences were assessed by one-way ANOVA followed by Tukey’s tests. ** *p* < 0.01, *** *p* < 0.001, and **** *p* < 0.0001.

Mitochondrial dysfunction plays a central role in NLRP3 inflammasome activation through multiple mechanisms, such as dissipation of mitochondrial membrane potential and the release of mitochondrial reactive oxygen species (mtROS) [25]. Next, we explored whether mitochondrial impairment was involved in ETEC-triggered pyroptosis in IECs. Mitochondrial membrane potential (MMP) was determined by JC-1. Results showed that the ETEC lysate significantly downregulated red fluorescence of JC-1 aggregate, but upregulated green fluorescence of JC-1 monomer. The ratio of JC-1 aggregate/monomer is much lower in ETEC primed Mode-k cells which indicated a significant decrease of MMP (Figure 6A). Dysfunction of MMP impairs the homeostasis of mitochondria and promotes mtROS production [26]. We next detected the fluorescence of MitoSox, mitochondrial superoxide anion radical (O_2_•^−^). ETEC lysate enhanced the mtROS levels by showing a significant increase of red fluorescence (Figure 6B). In addition, we also found that ETEC promoted the cytosolic release of mitochondrial DNA (mtDNA) and increased cytosolic cytochrome C (Cyt c) (Figure 6C,D). To further investigate whether ER stress response participated in ETEC-induced mitochondrial damage, we co-treated TUDCA with ETEC lysate in Mode-k cells. TUDCA treatment significantly reversed the ratio of JC-1 aggregate/monomer and decreased the mtROS production caused by ETEC lysate (Figure 6E,F). Therefore, our results demonstrated that ETEC impaired mitochondrial integrity and functions by activating ER stress response.

### 3.6. ETEC Activates cGAS/STING Signaling in IECs

**Figure 7:**
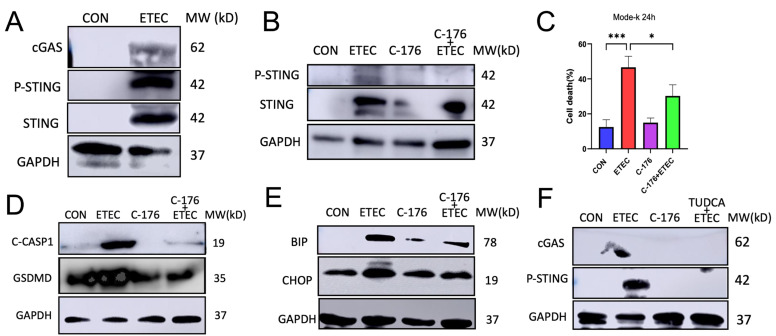
ETEC lysate activates STING in Mode-k cells. ETEC lysate was used to treat Mode-k cells for 24 h. (**A**) The expressions of cGAS, phosphorylated STING, and total STING were evaluated (n = 3). Mode-k cells were pre-treated with C-176 (2 μM) for 3 h and removed. Then, pre-treated Mode-k cells were treated with ETEC lysate for 24 h. (**B**) The expressions of phosphorylated STING and total STING were evaluated (n = 3). (**C**) Cell death was analyzed by CCK-8 assay. (**D**) The expressions of C-CASP1 and N-GSDMD were evaluated (n = 3). (**E**) The expressions of BIP and CHOP were evaluated (n = 3). ETEC lysate was used to treat Mode-k cells alone or in combination of TUDCA (800 μM) for 24 h. (**F**) The expressions of cGAS and phosphorylated STING were evaluated (n = 3). Data were presented as Mean ± SD. Differences were assessed by one-way ANOVA followed by Tukey’s tests. * *p* < 0.05 and *** *p* < 0.001.

The cyclic GMP-AMP synthase (cGAS) and the cyclic GMP-AMP receptor stimulator of interferon genes (STING) signaling, is an evolutionarily conserved defense mechanism that detects pathogenic DNA and triggers innate immune defense [27,28]. Recent studies revealed that mitochondrial damage associated ROS release switched on cGAS-STING signaling and promoted pyroptosis [19,29,30]. Our previous results indicated that ETEC damaged mitochondria and promoted mtROS production. We hypothesized that ETEC may activate cGAS/STING signaling and the activation contributed to ETEC-induced pyroptosis in IECs. Results demonstrated that ETEC lysate activated cGAS/STING signaling by upregulation of cGAS, phospho-STING and total STING in Mode-k cells (Figure 7A). C-176 is a specific STING inhibitor [31]. C-176 pre-treatment alleviated ETEC lysate-induced activation of STING (Figure 7B). More importantly, pre-treatment of C-176 rescued ETEC-induced cell death (Figure 7C) and inhibited the pyroptosis by blocking the activation of Caspase-1 and GSDMD in Mode-k cells (Figure 7D). Taken together, our results indicated that ETEC activated cGAS/STING signaling which contributed to ETEC-induced pyroptosis.

STING is an ER-associated membrane protein [32]. Activation of STING disrupted calcium homeostasis, which subsequently induced ER stress and activated UPR [32,33]. Both ER stress response and STING activation were involved in ETEC-induced IEC pyroptosis. We next investigated whether ETEC-induced STING activation triggered ER stress in Mode-k cells. Our results demonstrated that the elevation of the ER stress response markers, BIP and CHOP, was blocked by treating with C-176, indicating that STING activation contributed to ETEC-induced ER stress response (Figure 7E). Additionally, TUDCA treatment also attenuated ETEC-induced STING activation (Figure 7F). It suggested attenuation of ER stress response also inhibited STING activation. These findings suggest that a crosstalk between STING activation and ER stress response contributes to ETEC induced pyroptosis.

## 4. Discussion

**Figure 8:**
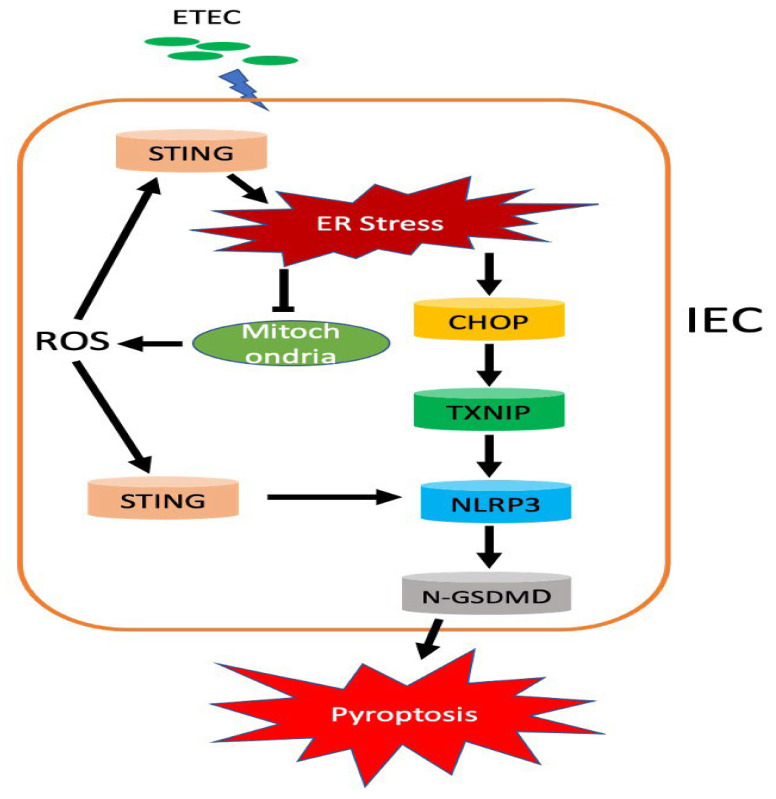
A model for ETEC to induce pyroptosis in IECs. ETEC activated ER stress, leading to upregulation of CHOP/TXNIP axis and impairment of mitochondria, which results in NLRP3 inflammasome/GSDMD-executed pyroptosis and ROS production in IECs, respectively. ROS activated STING, which further induced ER stress in IECs.

ETEC remains the most common cause of *E. coli* diarrhea in farm animals [34]. The high efficiency of infection makes ETEC easy to transmit in farm animals. Once establishing colonization in the intestine, ETEC can quickly multiply and secrete enterotoxins. These assaults activate inflammatory response to recruit immune cells and promote cell death of IECs. The IECs are the primary barrier between host cells and foreign pathogens. Aberrant increase of IECs’ cell death is a characteristic of infectious intestinal diseases [35]. Pyroptosis, as an inflammatory type of programmed cell death (PCD), plays a critical role in the host’s defense to microbial infections [36]. During pyroptotic process, two primary functions, selective release of processed cytokines and cell death, promote the elimination of a replicative niche of the pathogen [37]. However, chronic pyroptosis destructs the integrity of IECs. The resultant inflammation is considered as a major contribution to the pathogenesis of infectious gastrointestinal diseases [35,38,39]. It is crucial to understand the precise mechanisms that involve in the interplay between ETEC infection and pyroptosis. In our study, ETEC infection induced cell death of IECs by activating NLRP3/Caspase-1/GSDMD mediated pyroptosis both in vitro and in vivo. The increased cytokines were another indicative of pyroptosis. However, it is unclear whether release of cytokines was intestine-specific. ER stress response contributed to ETEC-induced pyroptosis of IECs. Activation of ER stress triggered mitochondrial impairment, facilitating the production of mtROS. At the same time, ER stress also activated STING, which promoted the NLRP3 inflammasome formation and pyroptosis of IECs (Figure 8).

ER stress response has been found that affects NLRP3 inflammasome activation and pyroptosis through multiple effects including the UPR signal transduction, calcium metabolism, and ROS production. The UPR is initiated by the activation of three ER transmembrane proteins, ATF6, IRE1 and PERK. TXNIP, a downstream factor of UPR, plays essential role in activating NLRP3 and promoting the formation of NLRP3 inflammasome complex by directly interacting with NLRP3 [23]. CHOP is an important convergent factor of the three branches of UPR, the elevation of which promotes the overexpression of TXNIP, and ultimately activates the NLRP3 inflammasome [18,23,40,41]. On the other hand, bacterial infections trigger ER stress response and activate UPR in mammalian cells [42]. ER provides a suitable niche for the intracellular survival and proliferation of intracellular bacteria, which results ER stress and induction of UPR [42]. Meanwhile, bacterial virulence factors, such as LPS and cytotoxins, also trigger ER stress and related cell death. For example, the shiga toxin 1 from enteric pathogens Shigella dysenteriae serotype 1 increased the activation of all three ER stress sensors and triggered apoptosis in epithelial cells [43]. The modulation of ER stress benefits the bacterial infections, but at the same time alters the homeostasis of ER, which promotes the pathogenesis by inducing inflammation and cell death [42,44]. In our study, ER stress and UPR that caused by ETEC infection was an important contributor to ETEC-mediated pyroptosis of IECs. Attenuation of ER stress by TUDCA significantly alleviated ETEC-mediated pyroptosis by inhibiting the activation of NLRP3 inflammasome. During the process, CHOP-mediated TXNIP upregulation contributed to the activation of NLRP3 inflammasome and subsequently pyroptosis. It suggests that targeting the ER stress or UPR signaling may provide a valuable therapeutic for ETEC-related intestinal infectious diseases.

Mitochondria function as a signaling center for a broad range of cellular activities, including inflammation, immune response and PCD [45,46]. As the cell powerhouse, mitochondria are responsible to produce ATP through oxidative phosphorylation. During the process, superoxide, the most important and dangerous mtROS is generated, whose abnormal increment induces cell injury via oxidative stress [47]. Mitochondrial damage associated cytosolic leakage of mitochondrial DNA (mtDNA) and mtROS induced NLRP3 inflammasome-mediated pyroptosis [48]. In addition, mitochondria-generated ROS also promoted S-palmitoylation of GSDMD, which activated GSDMD and caused pyroptosis [49]. On the other hand, activated GSDMD perforated mitochondria and damaged inner and outer mitochondrial membranes, accelerating pyroptosis [50]. Bacterial infections induce mitochondrial damage through virulence factors. For example, enteropathogenic *Escherichia coli* disrupted mitochondrial morphology, perturbed calcium homeostasis and triggered cell death by secreting T3SS effectors Map and EspF [51]. In our studies, ETEC impaired mitochondria and promoted the overproduction of mtROS that contributed to ETEC-mediated NLRP3 inflammasome activation and pyroptosis. TUDCA alleviated ETEC-caused mitochondrial damage, which suggested inhibition of ER stress may restored the mitochondrial functions in this process.

STING, also known as transmembrane protein 173 (TMEM173), is a multifunctional regulator of various types of PCDs, which has been implicated in the inflammatory damage of various diseases [19,32,52]. STING was demonstrated to interact and phosphorylate Type-I interferons (IFN) regulatory factor 3 (IRF3), which increased the expression of NLRP3, leading to inflammation and pyroptosis [53,54]. Inhibition of STING ameliorated NLRP3-GSDMD mediated pyroptosis [53,54]. In addition, STING was also reported to trigger calcium dependent GSDMD activation in bacterial sepsis [32]. Mitochondrial impairment in response to stress promotes the release of mitochondrial DNA (mtDNA) and excessive ROS, which are pivotal factors that activate STING signaling pathway [48,55]. Our results demonstrated that ETEC infection activated STING signaling pathway. Inhibition of STING by C-176 protected IECs against ETEC-induced pyroptosis. Moreover, STING is an ER-localized membrane protein. STING facilitated *E. coli* infection induced ER stress by promoting ER calcium release [32]. It suggests that STING is an upstream factor of ER stress during bacterial infections. Our results also indicated that inhibition of STING attenuated ETEC infection induced ER stress, which suggested that ETEC infection triggered pyroptosis by activating STING-ER stress-NLRP3-GSDMD axis. Interestingly, our results also demonstated that attenuation of ER stress ameliorated STING activation. ER stress impaired mitochondria which resulted ROS production and cytosolic release of mtDNA, critical factors that activated STING. Removal of ER stress restored the integrity of mitochondria, which attenuated STING activation. It suggested that STING activation and ER stress formed a positive feedback loop. ER stress activated STING by impairing mitochondria. Meanwhile, the STING activation promoted ER stress response.

## 5. Conclusions

This study demonstrates that ETEC infection activated NLRP3 inflammasome and induced pyroptosis of IECs in mice by triggering ER stress response. Mechanistically, ER stress impaired mitochondria to promote the generation of ROS and cytosolic release of mtDNA and Cyt c, while concurrently to activate STING to facilitate pyroptosis. It suggests that ER stress plays a critical role in ETEC-induced pyroptosis.

## Figures and Tables

**Table 1:** Primers used for qPCR amplification in this study.

Gene	Primer Set	Sequence (5′–3′)
NLRP3	Forward	GCCTCGGCCGCCTTTG
	Reverse	TCGATGTCCTTGGCGGAAAA
GAPDH	Forward	GAAAGCCTGCCGGTGACTAA
	Reverse	GCCCAATACGCACAAATCAGAG
TXNIP	Forward	CAAGGGTCTCAGCAGTGCAAAC
	Reverse	AAGCTCGAAGCCGAACTTGTACTC
STING	Forward	GGGTTTGGGGGCATCTTGAAA
	Reverse	AAAGGGCAGACAGCAGTCACA
cGAS	Forward	AGCTACCAAGGTGCTGTCAA
	Reverse	CCACGGTGACATCTGTATCTTTG
CASP1	Forward	AATACAACCACTCGTACACGTCTTG
	Reverse	ATCCTCCAGCAGCAACTTCATTTC
GSDMD	Forward	CCATCGGCCTTTGAGAAAGTG
	Reverse	ACACATGAATAACGGGGTTTCC
BIP	Forward	TCATCGGACGCACTTGGAA
	Reverse	CAACCTTGAATGGCAAGA
CHOP	Forward	GTCCCTAGCTTGGCTGACAGA
	Reverse	TGGAGAGCGAGGGCTTTG
PERK	Forward	GCCACUUUGAACUUCGGUAUA
	Reverse	UAUACCGAAGUUCAAAGUGGC

**Table 2:** Primers used for cytosolic mtDNA detection in this study.

Gene	Primer Set	Sequence (5′–3′)
ND1	Forward	TATCTCAACCCTAGCAGAAA
	Reverse	TAACGCGAATGGGCCGGCTG
18S	Forward	GTAACCCGTTGAACCCCATT
	Reverse	CCATCCAATCGGTAGTAGCG

## Data Availability

Research data are stored in an institutional repository and will be shared upon reasonable request to the corresponding author.

## Supplementary Materials

The following supporting information can be downloaded at: https://www.mdpi.com/article/10.3390/biology14121653/s1, Figure S1: ETEC lysate induces pyroptosis in Mode-k cells; Figure S2: ETEC induces intestinal pyroptosis in vivo; Figure S3: Mode-k cells were stimulated with LPS (500 ng/mL) for 5.5 h, followed by ATP (5 mM) for 30 min; File S1: Full-length, uncropped Western Blots.
