# Supplementary material for: Enterotoxigenic Escherichia coli (ETEC) Infection Triggers Pyroptosis Through ER Stress Response-Mediated Mitochondrial Impairment and STING Activation in Intestinal Epithelial Cells
Source: Biology (Basel). 2025 Nov 23;14(12):1653. doi: 10.3390/biology14121653 (PMC12729993; doi:10.3390/biology14121653)
Supplement: Supplementary file 1 [file biology-14-01653-s001.zip › biology-3924616-supplementary-Figures 11.21.pdf]

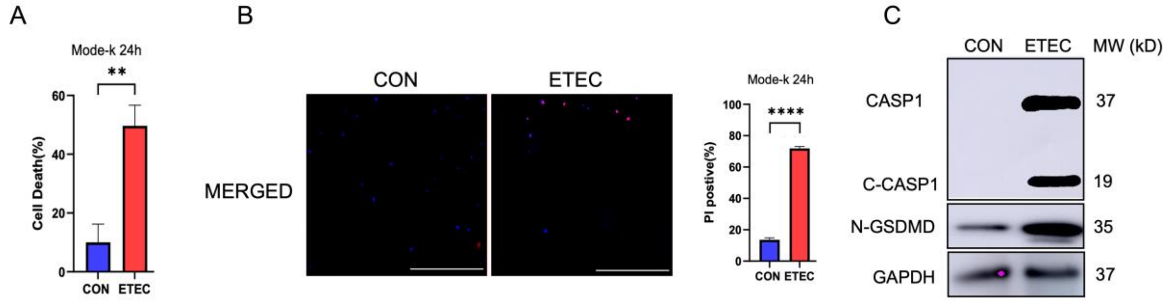

Figure S1. **ETEC lysate induces pyroptosis in Mode-k cells**. ETEC lysate was used to treat Mode-k cells for 24 hours. (A) Cell death was analyzed by CCK-8 assay. (B) Cell death was measured by quantifying the percentage of propidium iodide (PI) positive cells. (C) The expression of active CASP1 and N-GSDMD was evaluated Scale bar = 50 μm. Data were presented as Mean ± SD. Differences were assessed by one-way ANOVA followed by Tukey's tests. **p<0.01 and ****p<0.0001.

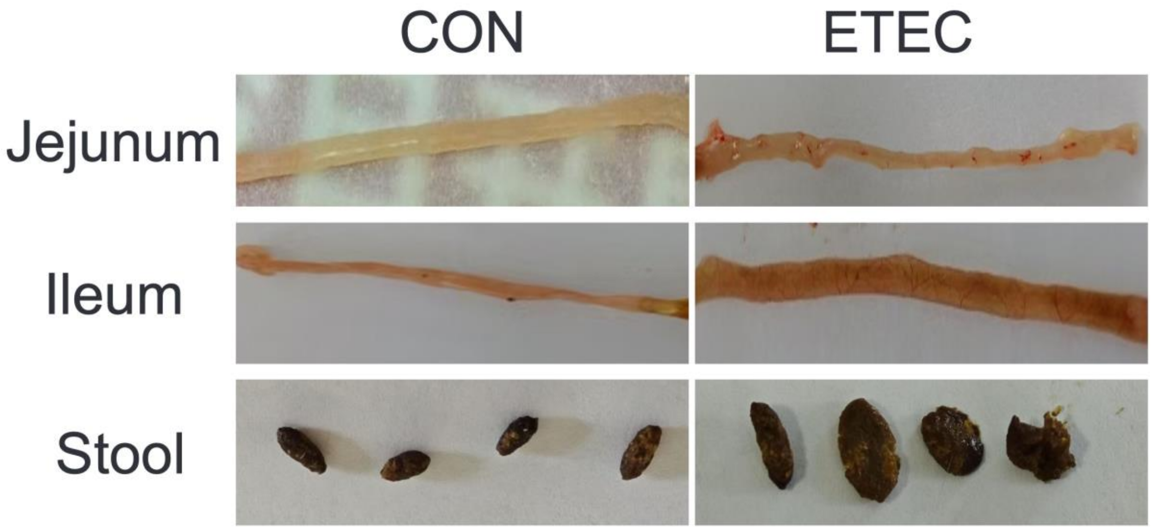

Figure S2. **ETEC induces intestinal pyroptosis *in vivo***. Streptomycin-pretreated C57BL/6mice were orally infected with 200 μL PBS (CON) or 1×10^{10} CFU/mL ETEC (ETEC) three times a day for 5 days. (A) Morphology of jejunum and ileum tissues. (B) The stools of CON and ETEC infected mice.

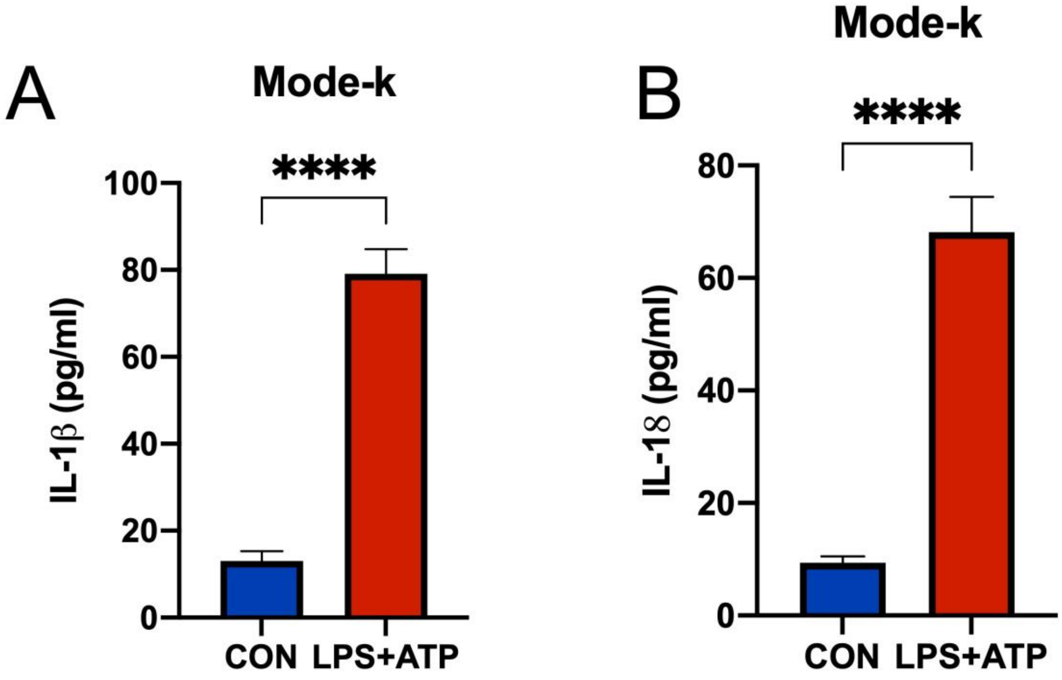

Figure S3. Mode-k cells were stimulated with LPS (500 ng/ml) for 5.5 hours, followed by ATP (5 mM) for 30 minutes. Cell culture supernatants were collected to analyze (A) IL-1β and (B) IL-18. Data were presented as Mean ± SD. Differences were assessed by unpaired t-test. ****$p<0.0001$.
