# Supplementary material for: Enterotoxigenic Escherichia coli (ETEC) Infection Triggers Pyroptosis Through ER Stress Response-Mediated Mitochondrial Impairment and STING Activation in Intestinal Epithelial Cells
Source: Biology (Basel). 2025 Nov 23;14(12):1653. doi: 10.3390/biology14121653 (PMC12729993; doi:10.3390/biology14121653)
Supplement: Supplementary file 1 [file biology-14-01653-s001.zip › biology-3924616-supplementary-Full-length, uncropped Western Blots 11.21.pdf]

FIGURE1E

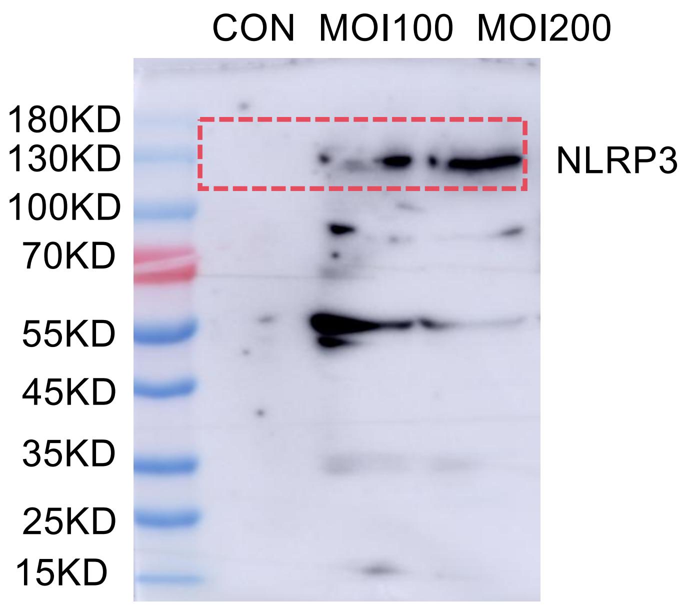

Figure 1E. Western blot membrane of NLRP3 (~110 kDa) protein detected with anti-NLRP3 (A5620, Abclonal; 1:1000) antibody. Gel-separated proteins were transferred to PVDF (0.45 μm pore size; Millipore) by wet electroblotting (200 mA for 90 min). Membranes, incubated with a HRP-conjugated secondary antibody (K1223, APExBIO; 1:5000), were developed with ECL (WBKLS0100, Millipore). #Weight marker (molecular weight in KD): Thermo ScientificTM/PageRulerTM Prestained Protein Ladder, 10 to 180 kDa; catalogue number: 26616.

FIGURE1E

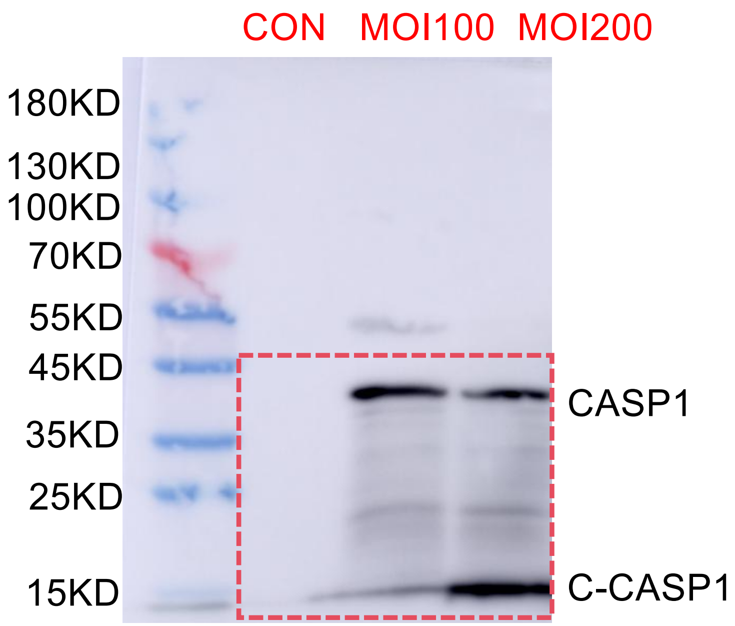

Figure 1E. Western blot membrane of Caspase-1 (CASP1) (~37 kDa) and cleaved-Caspase-1 (C-CASP1) (~19 kDa) protein detected with anti-Caspase-1 (AF5418, Affinity Bioscience; 1:1000) antibody. Gel-separated proteins were transferred to PVDF (0.45 µm pore size; Millipore) by wet electroblotting (200 mA for 90 min). Membranes, incubated with a HRP-conjugated secondary antibody (K1223, APExBIO; 1:5000), were developed with ECL (WBKLS0100, Millipore). #Weight marker (molecular weight in KD): Thermo ScientificTM/PageRulerTM Prestained Protein Ladder, 10 to 180 kDa; catalogue number: 26616.

FIGURE1E

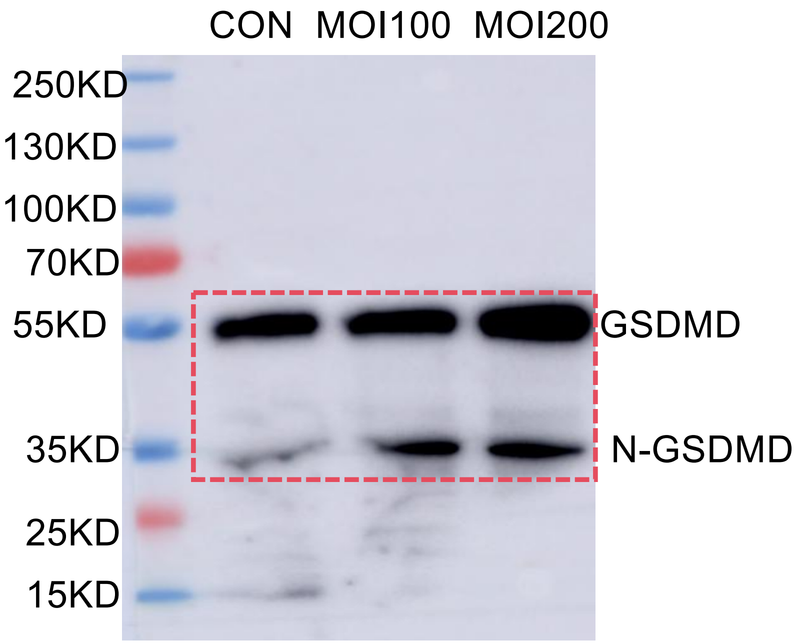

Figure 1E. Western blot membrane of gasderminD (GSDMD) (~55 kDa) and N terminal GSDMD (N-GSDMD) (~35 kDa) protein detected with anti-GSDMD (AF4012, Affinity Bioscience ; 1:1000) antibody. Gel-separated proteins were transferred to PVDF (0.45 µm pore size; Millipore) by wet electroblotting (200 mA for 90 min). Membranes, incubated with a HRP-conjugated secondary antibody (K1223, APExBIO; 1:5000), were developed with ECL (WBKLS0100, Millipore). #Weight marker (molecular weight in KD): Thermo ScientificTM/PageRulerTM Prestained Protein Ladder, 10 to 180 kDa; catalogue number: 26616.

FIGURE1E

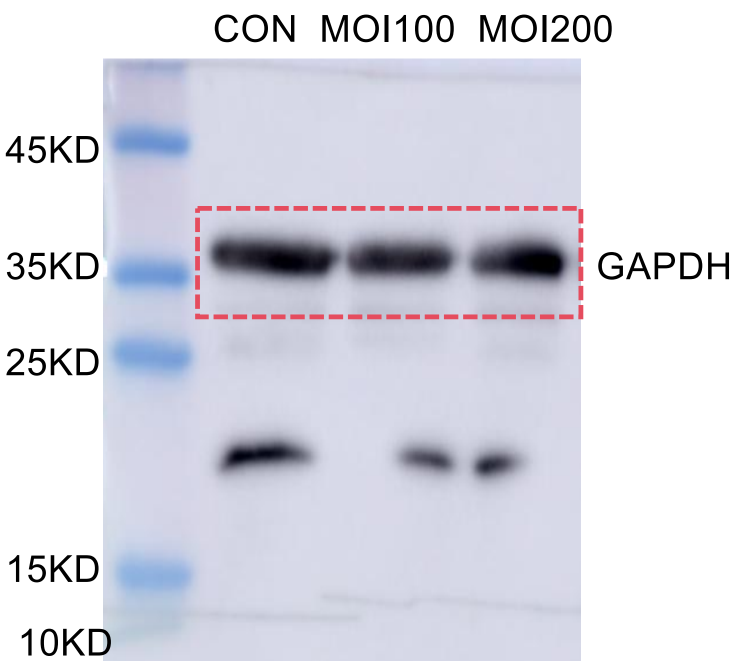

Figure 1E. Western blot membrane of GAPDH (~37 kDa) protein detected with anti-GAPDH (AF7021, Affinity Bioscience; 1:3000) antibody. Gel-separated proteins were transferred to PVDF (0.45 µm pore size; Millipore) by wet electroblotting (200 mA for 90 min). Membranes, incubated with a HRP-conjugated secondary antibody (K1223, APExBIO; 1:5000), were developed with ECL (WBKLS0100, Millipore). #Weight marker (molecular weight in KD): Thermo ScientificTM/PageRulerTM Prestained Protein Ladder, 10 to 180 kDa; catalogue number: 26616.

FIGURE2C

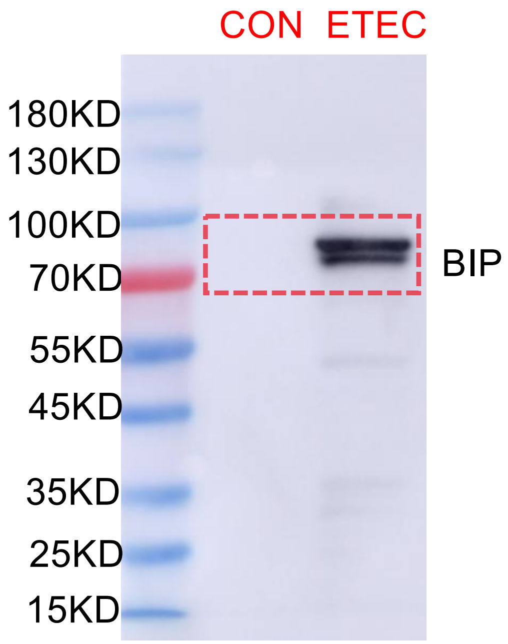

Figure 2C. Western blot membrane of BIP (~78 kDa) protein detected with anti-BIP (AF5366, Affinity Bioscience; 1:1000) antibody. Gel-separated proteins were transferred to PVDF (0.45 µm pore size; Millipore) by wet electroblotting (200 mA for 90 min). Membranes, incubated with a HRP-conjugated secondary antibody (K1223, APExBIO; 1:5000), were developed with ECL (WBKLS0100, Millipore). #Weight marker (molecular weight in KD): Thermo ScientificTM/PageRulerTM Prestained Protein Ladder, 10 to 180 kDa; catalogue number: 26616.

FIGURE2C

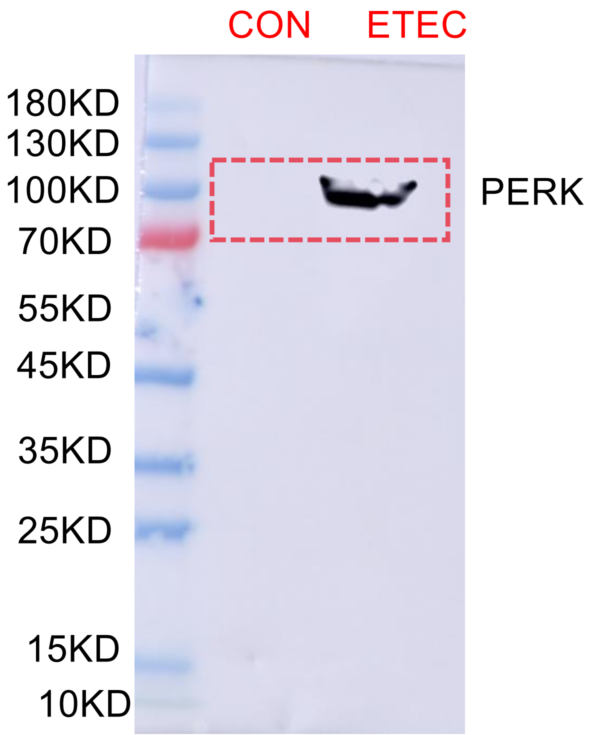

Figure 2C. Western blot membrane of PERK (~124 kDa) protein detected with anti-PERK (A18196, Abclonal; 1:1000) antibody. Gel-separated proteins were transferred to PVDF (0.45 µm pore size; Millipore) by wet electroblotting (200 mA for 90 min). Membranes, incubated with a HRP-conjugated secondary antibody (K1223, APExBIO; 1:5000), were developed with ECL (WBKLS0100, Millipore). #Weight marker (molecular weight in KD): Thermo ScientificTM/PageRulerTM Prestained Protein Ladder, 10 to 180 kDa; catalogue number: 26616.

FIGURE2C

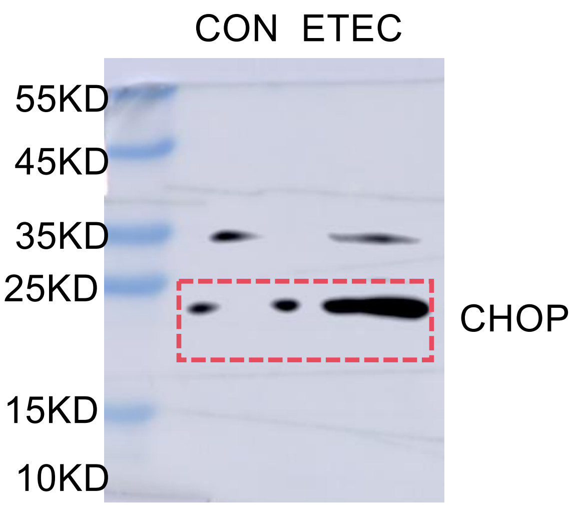

Figure 2C. Western blot membrane of CHOP (~19 kDa) protein detected with anti-CHOP (AF6277, Affinity Bioscience; 1:1000) antibody. Gel-separated proteins were transferred to PVDF (0.45 µm pore size; Millipore) by wet electroblotting (200 mA for 90 min). Membranes, incubated with a HRP-conjugated secondary antibody (K1223, APExBIO; 1:5000), were developed with ECL (WBKLS0100, Millipore). #Weight marker (molecular weight in KD): Thermo ScientificTM/PageRulerTM Prestained Protein Ladder, 10 to 180 kDa; catalogue number: 26616.

FIGURE2C

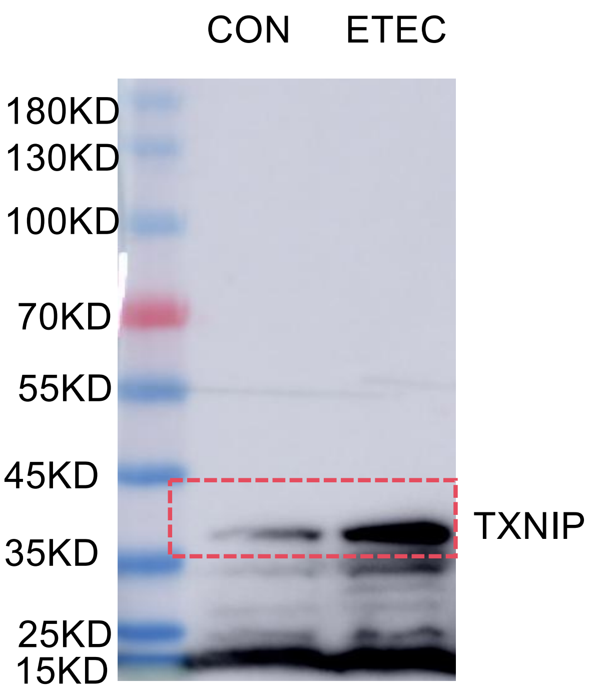

Figure 2C. Western blot membrane of TXNIP (~37 kDa) protein detected with anti-TXNIP (AF8277, Beyotime; 1:1000) antibody. Gel-separated proteins were transferred to PVDF (0.45 µm pore size; Millipore) by wet electroblotting (200 mA for 90 min). Membranes, incubated with a HRP-conjugated secondary antibody (K1223, APExBIO; 1:5000), were developed with ECL (WBKLS0100, Millipore). #Weight marker (molecular weight in KD): Thermo ScientificTM/PageRulerTM Prestained Protein Ladder, 10 to 180 kDa; catalogue number: 26616.

FIGURE2C

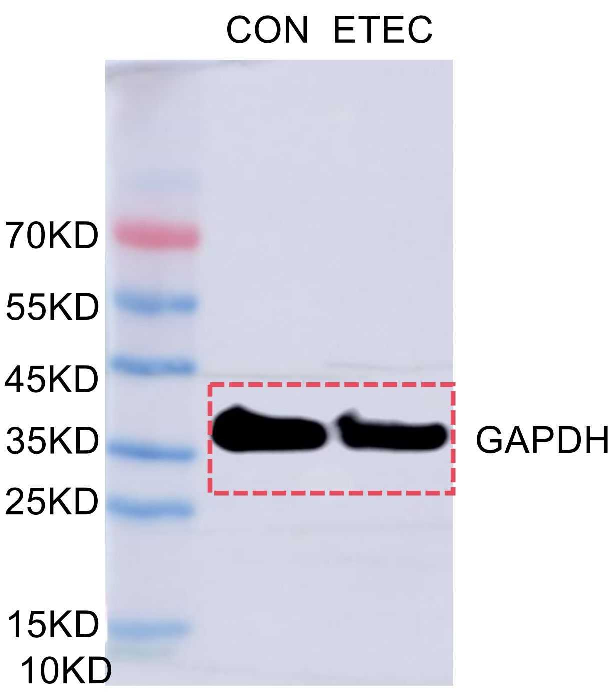

Figure 2C. Western blot membrane of GAPDH (~37 kDa) protein detected with anti-GAPDH (AF7021, Affinity Bioscience; 1:3000) antibody. Gel-separated proteins were transferred to PVDF (0.45 µm pore size; Millipore) by wet electroblotting (200 mA for 90 min). Membranes, incubated with a HRP-conjugated secondary antibody (K1223, APExBIO; 1:5000), were developed with ECL (WBKLS0100, Millipore). #Weight marker (molecular weight in KD): Thermo ScientificTM/PageRulerTM Prestained Protein Ladder, 10 to 180 kDa; catalogue number: 26616.

FIGURE3D

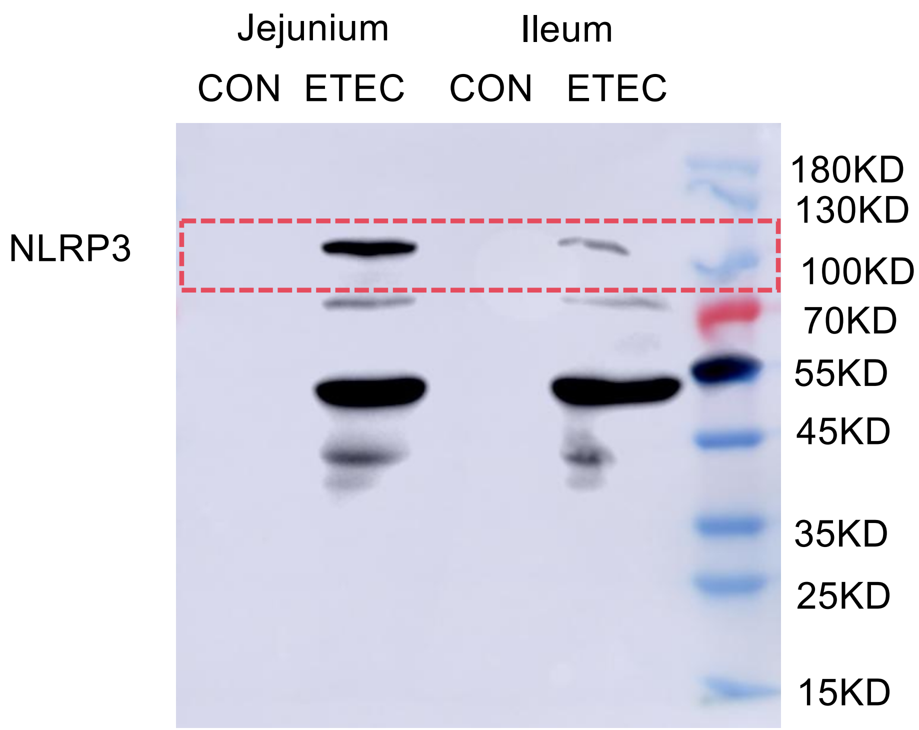

Figure 3D. Western blot membrane of NLRP3 (~110 kDa) protein detected with anti-NLRP3 (A5620, Abclonal; 1:1000) antibody. Gel-separated proteins were transferred to PVDF (0.45 µm pore size; Millipore) by wet electroblotting (200 mA for 90 min). Membranes, incubated with a HRP-conjugated secondary antibody (K1223, APExBIO; 1:5000), were developed with ECL (WBKLS0100, Millipore). #Weight marker (molecular weight in KD): Thermo ScientificTM/PageRulerTM Prestained Protein Ladder, 10 to 180 kDa; catalogue number: 26616.

FIGURE3D

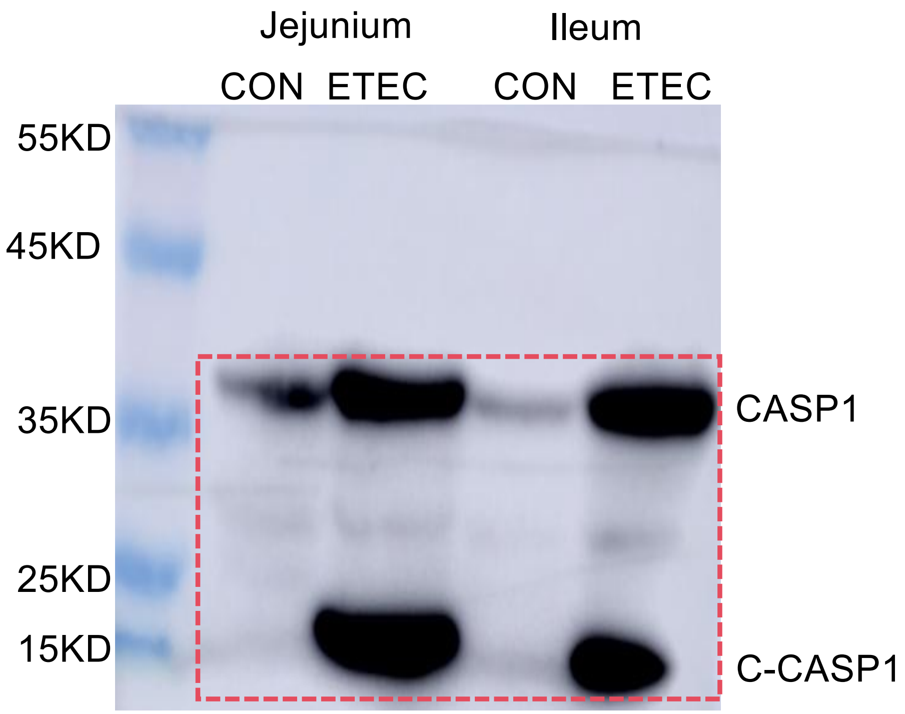

Figure 3D. Western blot membrane of Caspase-1 (CASP1) (~37 kDa) and cleaved-Caspase-1 (C-CASP1) (~19 kDa) protein detected with anti-Caspase-1 (AF5418, Affinity Bioscience; 1:1000) antibody. Gel-separated proteins were transferred to PVDF (0.45 µm pore size; Millipore) by wet electroblotting (200 mA for 90 min). Membranes, incubated with a HRP-conjugated secondary antibody (K1223, APExBIO; 1:5000), were developed with ECL (WBKLS0100, Millipore). #Weight marker (molecular weight in KD): Thermo ScientificTM/PageRulerTM Prestained Protein Ladder, 10 to 180 kDa; catalogue number: 26616.

FIGURE3D

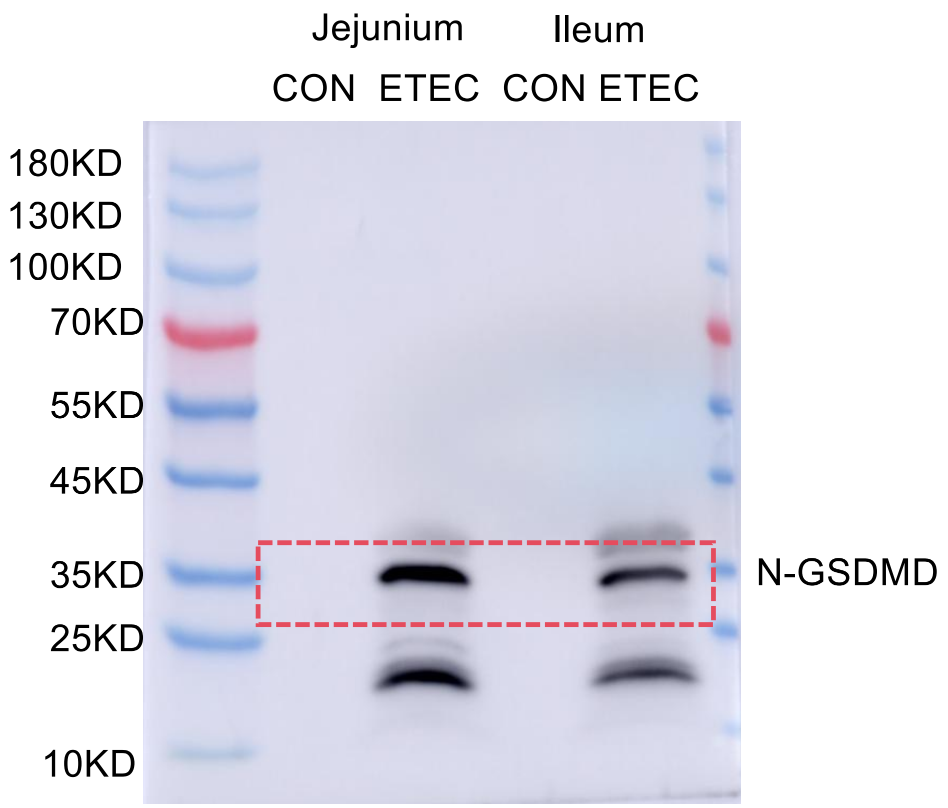

Figure 3D. Western blot membrane of N terminal GSDMD (N-GSDMD) (~35 kDa) protein detected with anti-GSDMD (AF4012, Affinity Bioscience ; 1:1000) antibody. Gel-separated proteins were transferred to PVDF (0.45 µm pore size; Millipore) by wet electroblotting (200 mA for 90 min). Membranes, incubated with a HRP-conjugated secondary antibody (K1223, APExBIO; 1:5000), were developed with ECL (WBKLS0100, Millipore). #Weight marker (molecular weight in KD): Thermo ScientificTM/PageRulerTM Prestained Protein Ladder, 10 to 180 kDa; catalogue number: 26616.

FIGURE3D

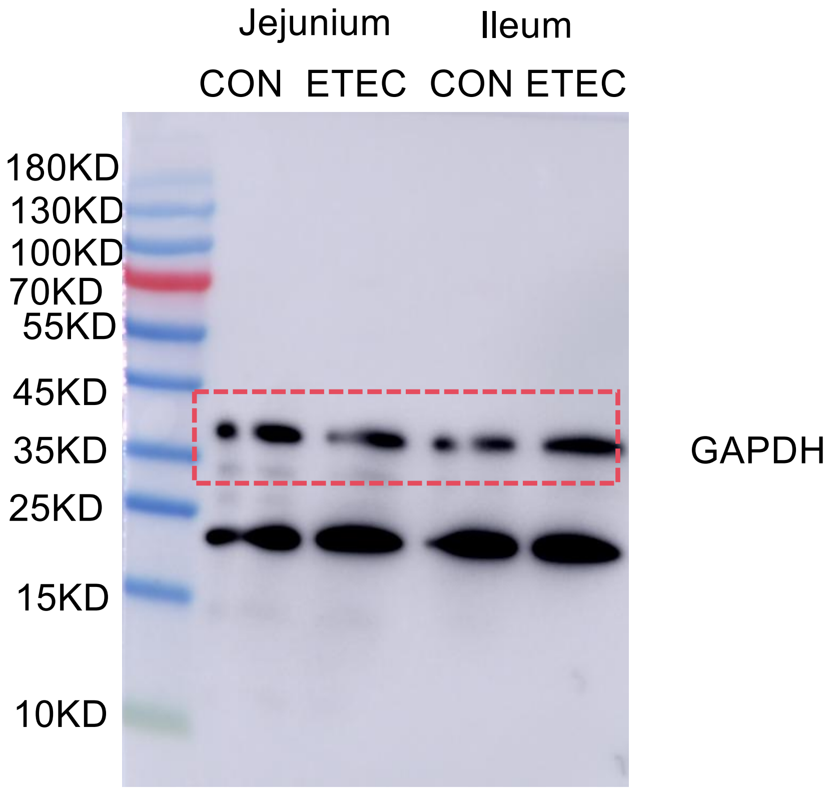

Figure 3D. Western blot membrane of GAPDH (~37 kDa) protein detected with anti-GAPDH (AF7021, Affinity Bioscience; 1:3000) antibody. Gel-separated proteins were transferred to PVDF (0.45 µm pore size; Millipore) by wet electroblotting (200 mA for 90 min). Membranes, incubated with a HRP-conjugated secondary antibody (K1223, APExBIO; 1:5000), were developed with ECL (WBKLS0100, Millipore). #Weight marker (molecular weight in KD): Thermo ScientificTM/PageRulerTM Prestained Protein Ladder, 10 to 180 kDa; catalogue number: 26616.

FIGURE3G

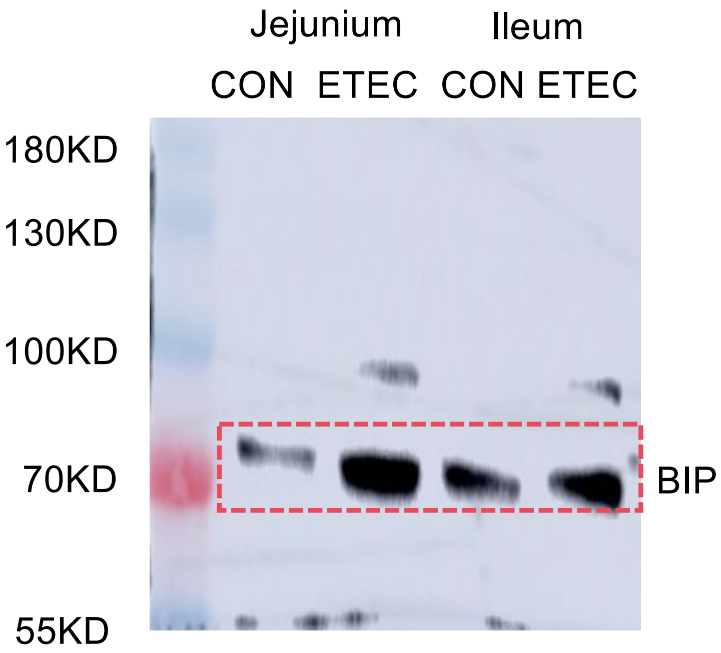

Figure 3G. Western blot membrane of BIP (~78 kDa) protein detected with anti-BIP (AF5366, Affinity Bioscience; 1:1000) antibody. Gel-separated proteins were transferred to PVDF (0.45 µm pore size; Millipore) by wet electroblotting (200 mA for 90 min). Membranes, incubated with a HRP-conjugated secondary antibody (K1223, APExBIO; 1:5000), were developed with ECL (WBKLS0100, Millipore). #Weight marker (molecular weight in KD): Thermo ScientificTM/PageRulerTM Prestained Protein Ladder, 10 to 180 kDa; catalogue number: 26616.

FIGURE3G

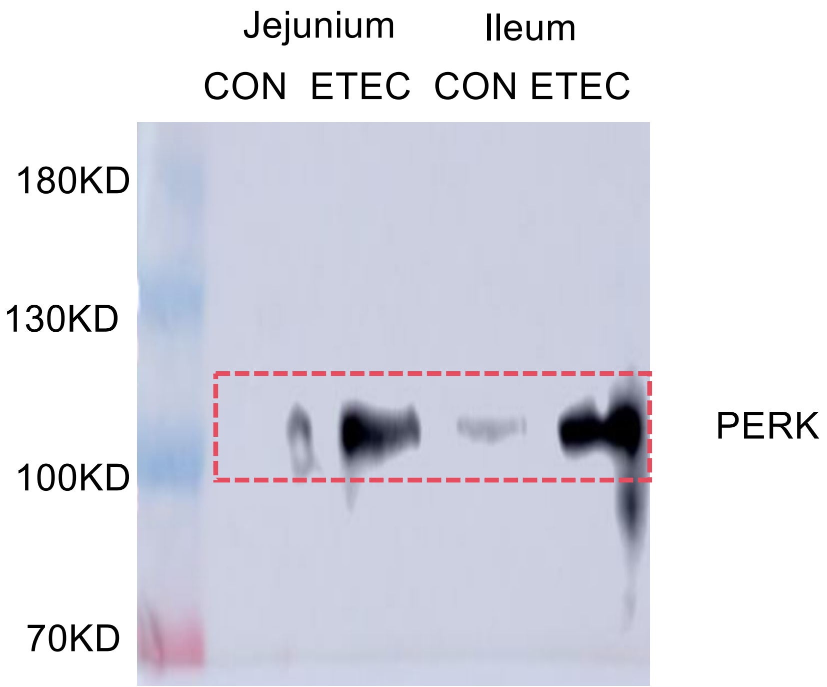

Figure 3G. Western blot membrane of PERK (~124 kDa) protein detected with anti-PERK (A18196, Abclonal; 1:1000) antibody. Gel-separated proteins were transferred to PVDF (0.45 µm pore size; Millipore) by wet electroblotting (200 mA for 90 min). Membranes, incubated with a HRP-conjugated secondary antibody (K1223, APExBIO; 1:5000), were developed with ECL (WBKLS0100, Millipore). #Weight marker (molecular weight in KD): Thermo ScientificTM/PageRulerTM Prestained Protein Ladder, 10 to 180 kDa; catalogue number: 26616.

FIGURE3G

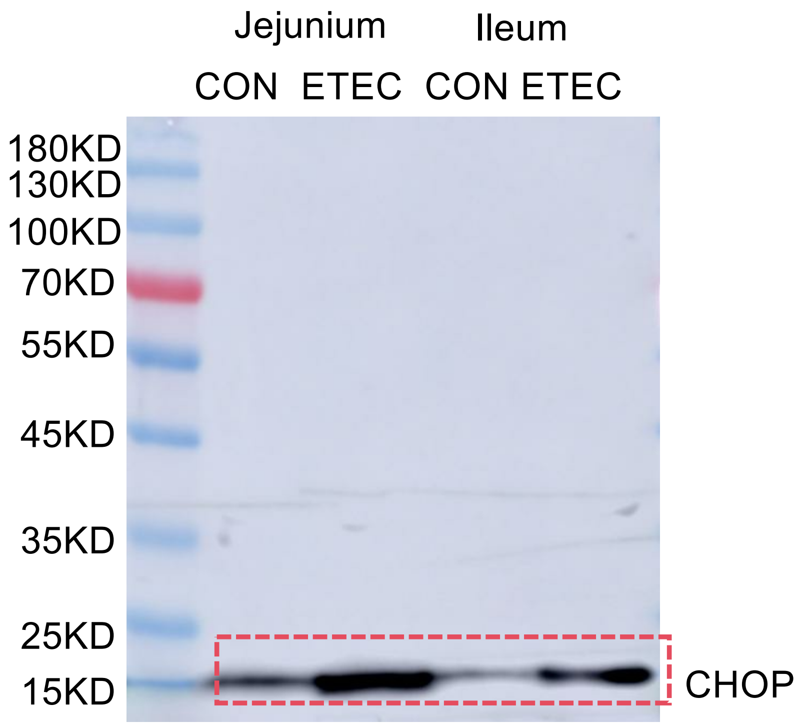

Figure 3G. Western blot membrane of CHOP (~19 kDa) protein detected with anti-CHOP (AF6277, Affinity Bioscience; 1:1000) antibody. Gel-separated proteins were transferred to PVDF (0.45 µm pore size; Millipore) by wet electroblotting (200 mA for 90 min). Membranes, incubated with a HRP-conjugated secondary antibody (K1223, APExBIO; 1:5000), were developed with ECL (WBKLS0100, Millipore). #Weight marker (molecular weight in KD): Thermo ScientificTM/PageRulerTM Prestained Protein Ladder, 10 to 180 kDa; catalogue number: 26616.

FIGURE3G

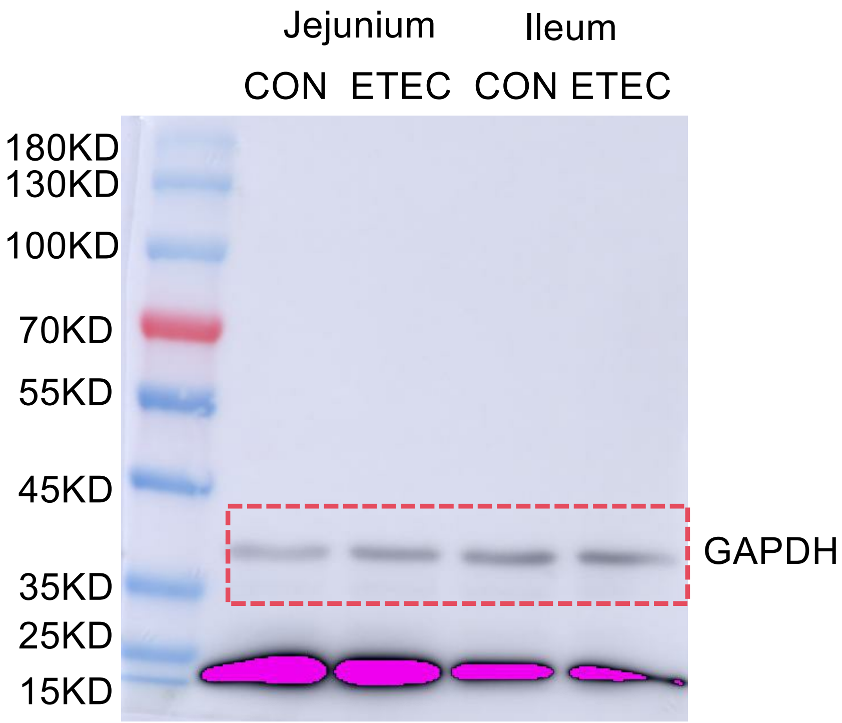

Figure 3G. Western blot membrane of GAPDH (~37 kDa) protein detected with anti-GAPDH (AF7021, Affinity Bioscience; 1:3000) antibody. Gel-separated proteins were transferred to PVDF (0.45 µm pore size; Millipore) by wet electroblotting (200 mA for 90 min). Membranes, incubated with a HRP-conjugated secondary antibody (K1223, APExBIO; 1:5000), were developed with ECL (WBKLS0100, Millipore). #Weight marker (molecular weight in KD): Thermo ScientificTM/PageRulerTM Prestained Protein Ladder, 10 to 180 kDa; catalogue number: 26616.

FIGURE4C

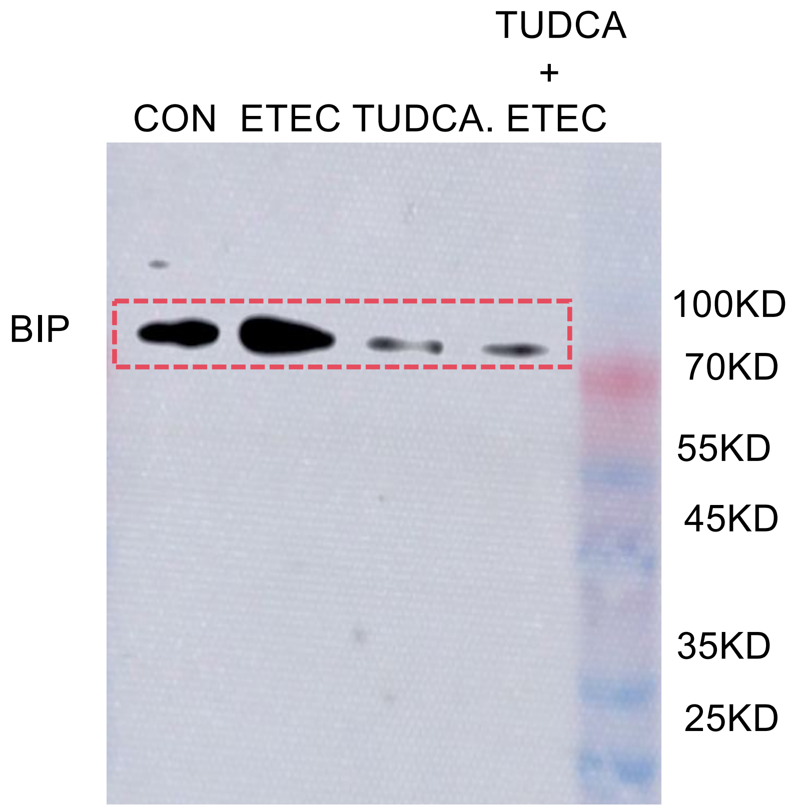

Figure 4C. Western blot membrane of BIP (~78 kDa) protein detected with anti-BIP (AF5366, Affinity Bioscience; 1:1000) antibody. Gel-separated proteins were transferred to PVDF (0.45 µm pore size; Millipore) by wet electroblotting (200 mA for 90 min). Membranes, incubated with a HRP-conjugated secondary antibody (K1223, APExBIO; 1:5000), were developed with ECL (WBKLS0100, Millipore). #Weight marker (molecular weight in KD): Thermo ScientificTM/PageRulerTM Prestained Protein Ladder, 10 to 180 kDa; catalogue number: 26616.

FIGURE4C

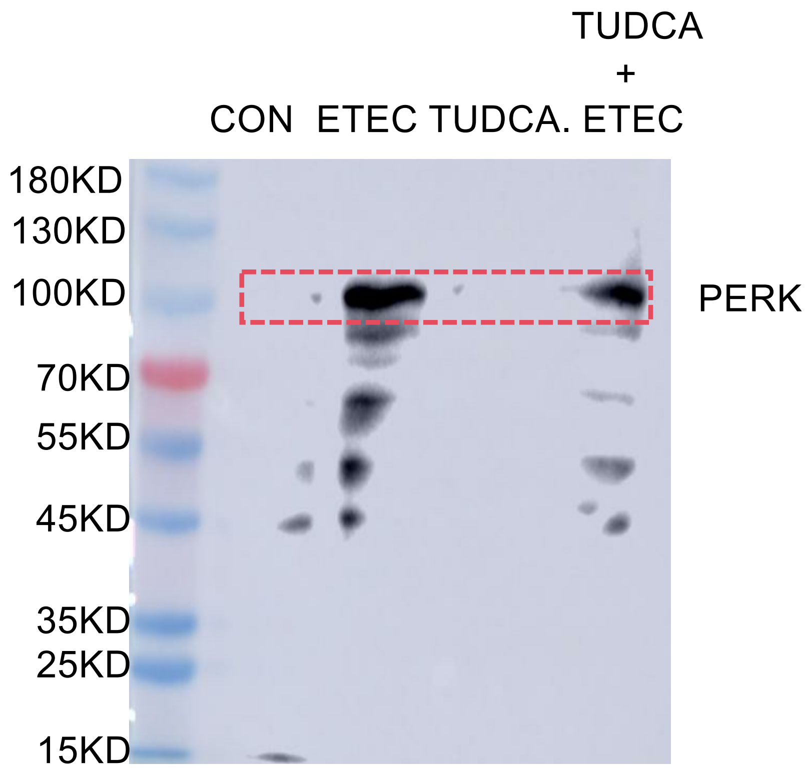

Figure 4C. Western blot membrane of PERK (~124 kDa) protein detected with anti-PERK (A18196, Abclonal; 1:1000) antibody. Gel-separated proteins were transferred to PVDF (0.45 µm pore size; Millipore) by wet electroblotting (200 mA for 90 min). Membranes, incubated with a HRP-conjugated secondary antibody (K1223, APExBIO; 1:5000), were developed with ECL (WBKLS0100, Millipore). #Weight marker (molecular weight in KD): Thermo ScientificTM/PageRulerTM Prestained Protein Ladder, 10 to 180 kDa; catalogue number: 26616.

FIGURE4C

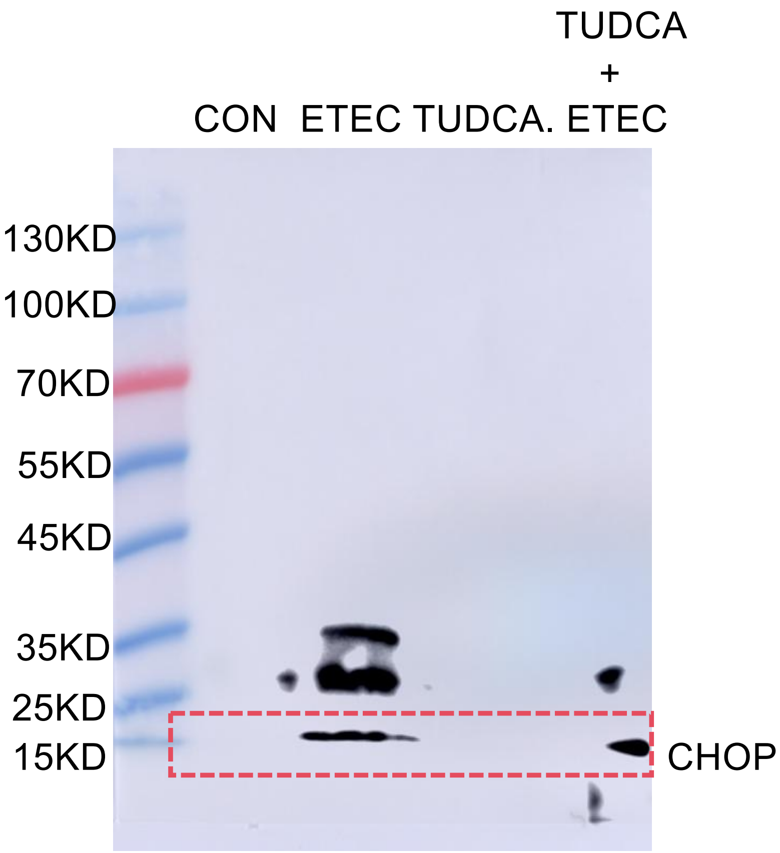

Figure 4C. Western blot membrane of CHOP (~19 kDa) protein detected with anti-CHOP (AF6277, Affinity Bioscience; 1:1000) antibody. Gel-separated proteins were transferred to PVDF (0.45 µm pore size; Millipore) by wet electroblotting (200 mA for 90 min). Membranes, incubated with a HRP-conjugated secondary antibody (K1223, APExBIO; 1:5000), were developed with ECL (WBKLS0100, Millipore). #Weight marker (molecular weight in KD): Thermo ScientificTM/PageRulerTM Prestained Protein Ladder, 10 to 180 kDa; catalogue number: 26616.

FIGURE4C

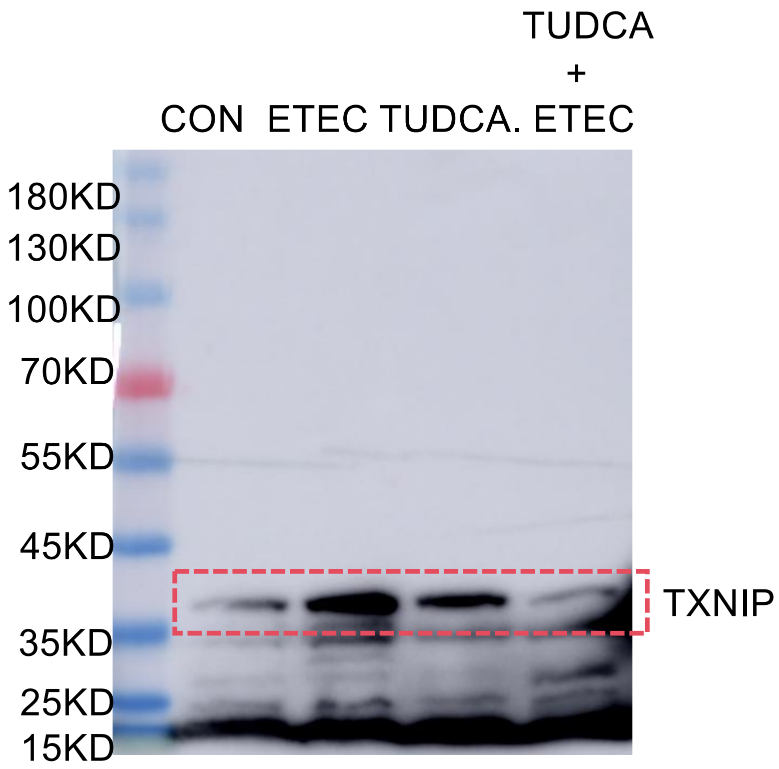

Figure 4C. Western blot membrane of TXNIP (~37 kDa) protein detected with anti-TXNIP (AF8277, Beyotime; 1:1000) antibody. Gel-separated proteins were transferred to PVDF (0.45 µm pore size; Millipore) by wet electroblotting (200 mA for 90 min). Membranes, incubated with a HRP-conjugated secondary antibody (K1223, APExBIO; 1:5000), were developed with ECL (WBKLS0100, Millipore). #Weight marker (molecular weight in KD): Thermo ScientificTM/PageRulerTM Prestained Protein Ladder, 10 to 180 kDa; catalogue number: 26616.

FIGURE4C

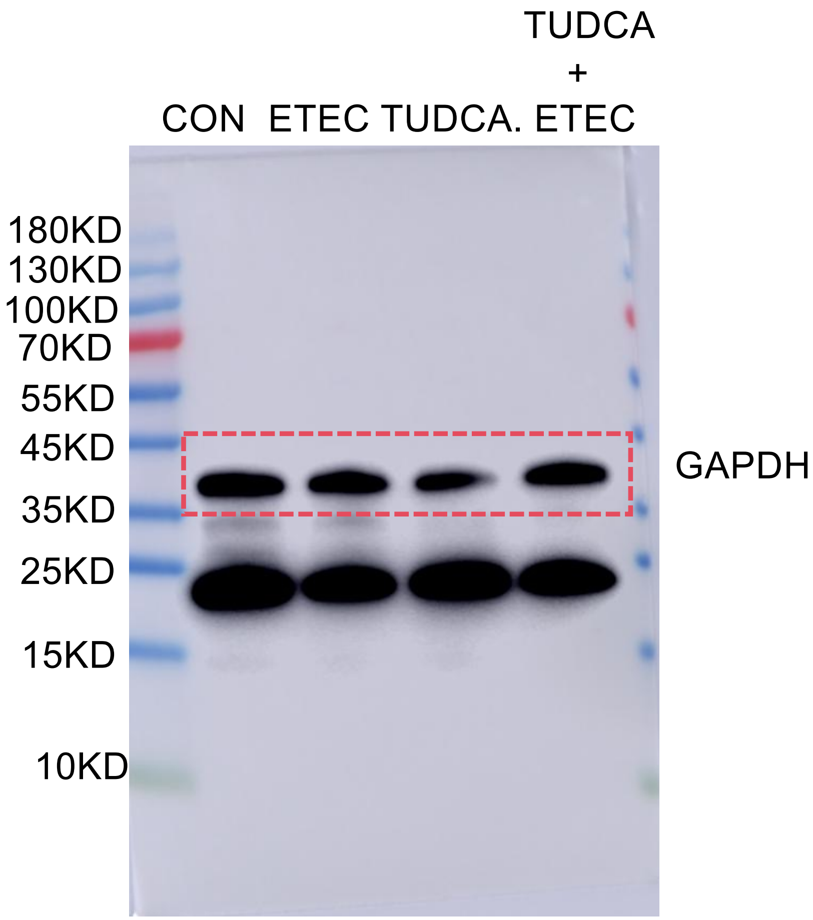

Figure 4C. Western blot membrane of GAPDH (~37 kDa) protein detected with anti-GAPDH (AF7021, Affinity Bioscience; 1:3000) antibody. Gel-separated proteins were transferred to PVDF (0.45 µm pore size; Millipore) by wet electroblotting (200 mA for 90 min). Membranes, incubated with a HRP-conjugated secondary antibody (K1223, APExBIO; 1:5000), were developed with ECL (WBKLS0100, Millipore). #Weight marker (molecular weight in KD): Thermo ScientificTM/PageRulerTM Prestained Protein Ladder, 10 to 180 kDa; catalogue number: 26616.

FIGURE4G

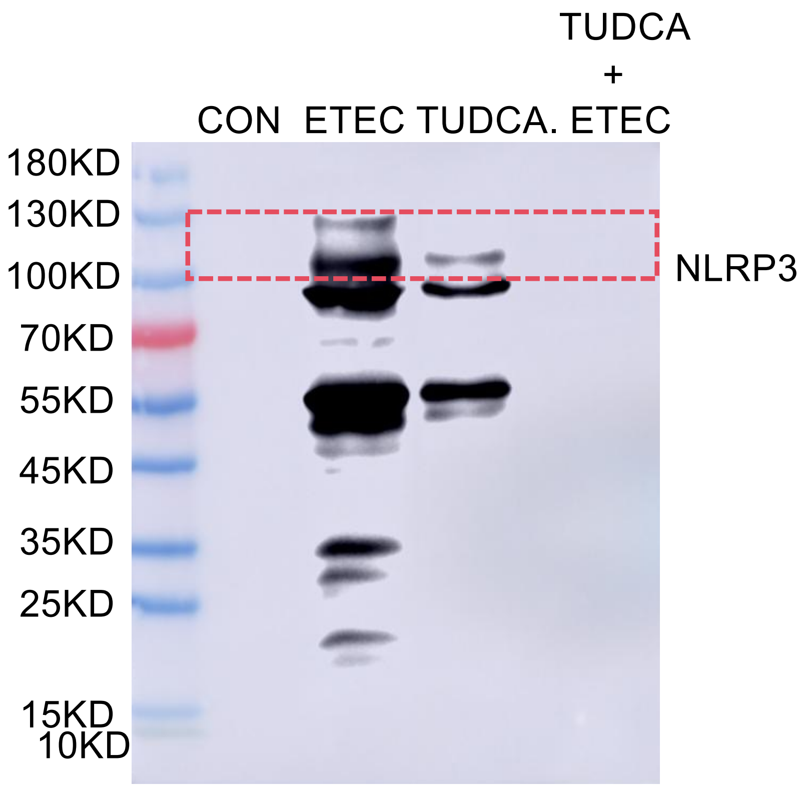

Figure 4G. Western blot membrane of NLRP3 (~110 kDa) protein detected with anti-NLRP3 (A5620, Abclonal; 1:1000) antibody. Gel-separated proteins were transferred to PVDF (0.45 µm pore size; Millipore) by wet electroblotting (200 mA for 90 min). Membranes, incubated with a HRP-conjugated secondary antibody (K1223, APExBIO; 1:5000), were developed with ECL (WBKLS0100, Millipore). #Weight marker (molecular weight in KD): Thermo ScientificTM/PageRulerTM Prestained Protein Ladder, 10 to 180 kDa; catalogue number: 26616.

FIGURE4G

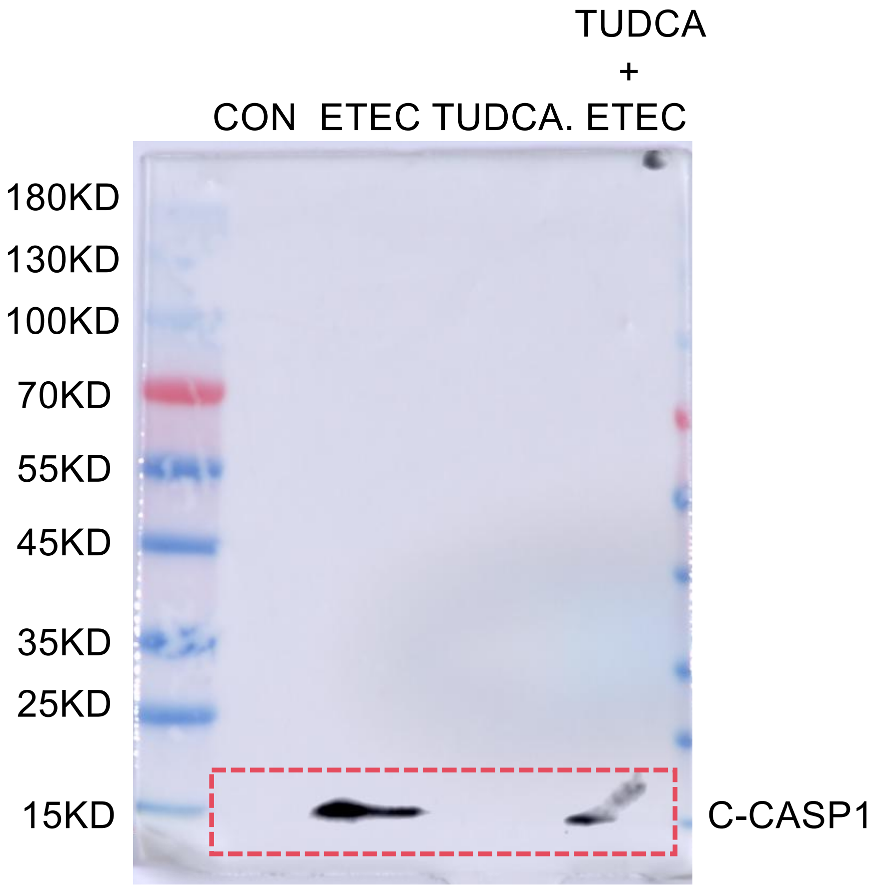

Figure 4G. Western blot membrane of cleaved-Caspase-1 (C-CASP1) (~19 kDa) protein detected with anti-Caspase-1 (AF5418, Affinity Bioscience; 1:1000) antibody. Gel-separated proteins were transferred to PVDF (0.45 μm pore size; Millipore) by wet electroblotting (200 mA for 90 min). Membranes, incubated with a HRP-conjugated secondary antibody (K1223, APExBIO; 1:5000), were developed with ECL (WBKLS0100, Millipore). #Weight marker (molecular weight in KD): Thermo ScientificTM/PageRulerTM Prestained Protein Ladder, 10 to 180 kDa; catalogue number: 26616.

FIGURE4G

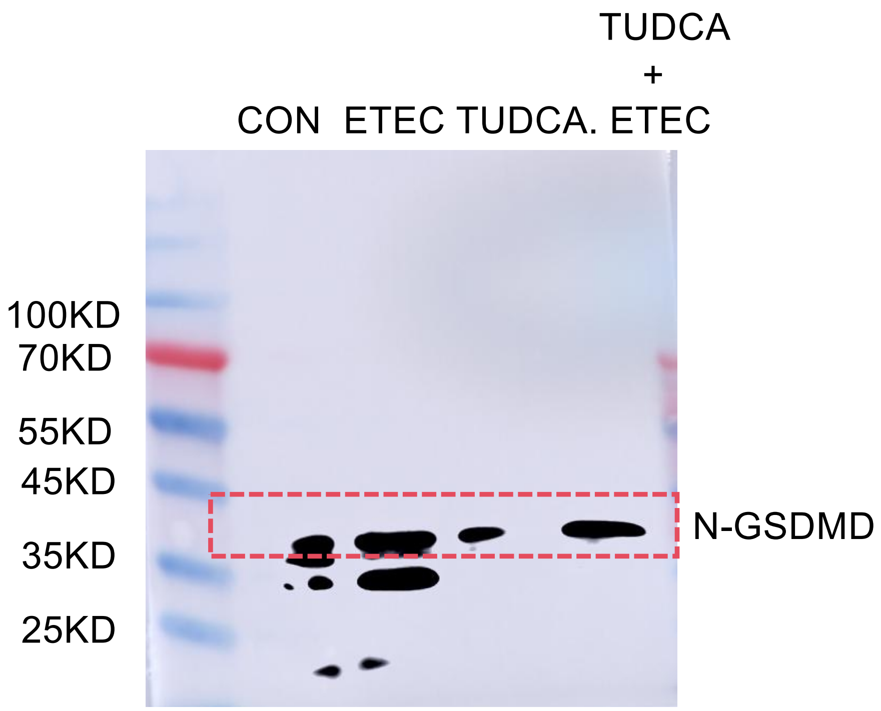

Figure 4G. Western blot membrane of N terminal GSDMD (N-GSDMD) (~35 kDa) protein detected with anti-GSDMD (AF4012, Affinity Bioscience ; 1:1000) antibody. Gel-separated proteins were transferred to PVDF (0.45 µm pore size; Millipore) by wet electroblotting (200 mA for 90 min). Membranes, incubated with a HRP-conjugated secondary antibody (K1223, APExBIO; 1:5000), were developed with ECL (WBKLS0100, Millipore). #Weight marker (molecular weight in KD): Thermo ScientificTM/PageRulerTM Prestained Protein Ladder, 10 to 180 kDa; catalogue number: 26616.

FIGURE4G

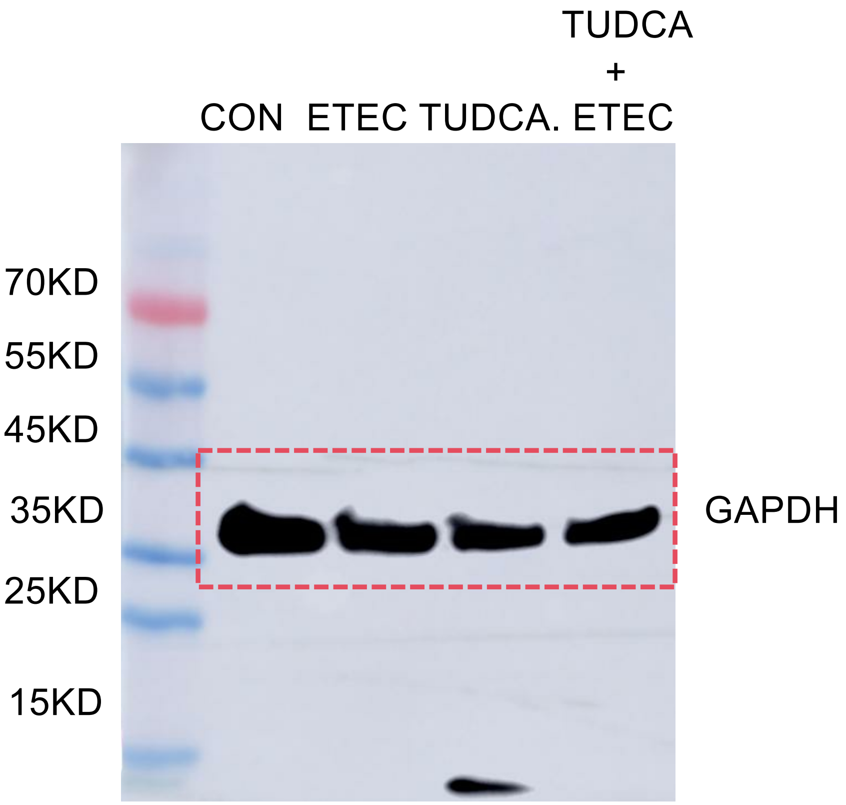

Figure 4G. Western blot membrane of GAPDH (~37 kDa) protein detected with anti-GAPDH (AF7021, Affinity Bioscience; 1:3000) antibody. Gel-separated proteins were transferred to PVDF (0.45 µm pore size; Millipore) by wet electroblotting (200 mA for 90 min). Membranes, incubated with a HRP-conjugated secondary antibody (K1223, APExBIO; 1:5000), were developed with ECL (WBKLS0100, Millipore). #Weight marker (molecular weight in KD): Thermo ScientificTM/PageRulerTM Prestained Protein Ladder, 10 to 180 kDa; catalogue number: 26616.

FIGURE5D

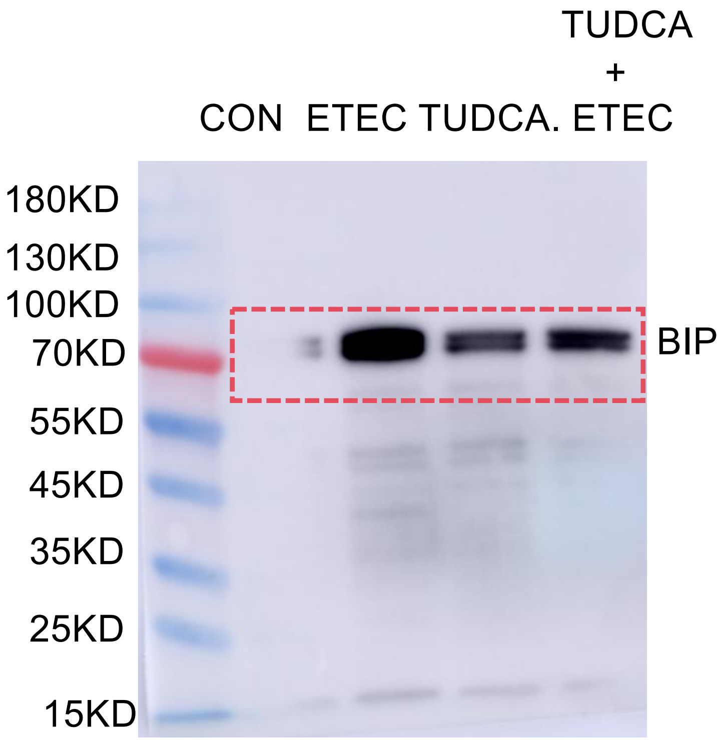

Figure 5D. Western blot membrane of BIP (~78 kDa) protein detected with anti-BIP (AF5366, Affinity Bioscience; 1:1000) antibody. Gel-separated proteins were transferred to PVDF (0.45 μm pore size; Millipore) by wet electroblotting (200 mA for 90 min). Membranes, incubated with a HRP-conjugated secondary antibody (K1223, APExBIO; 1:5000), were developed with ECL (WBKLS0100, Millipore). #Weight marker (molecular weight in KD): Thermo ScientificTM/PageRulerTM Prestained Protein Ladder, 10 to 180 kDa; catalogue number: 26616.

FIGURE5D

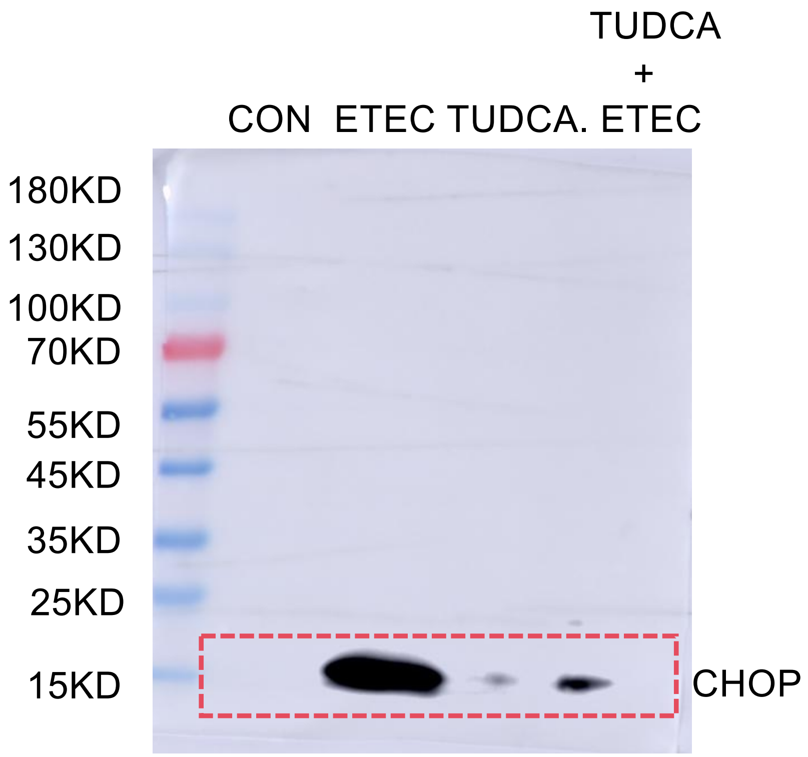

Figure 5D. Western blot membrane of CHOP (~19 kDa) protein detected with anti-CHOP (AF6277, Affinity Bioscience; 1:1000) antibody. Gel-separated proteins were transferred to PVDF (0.45 µm pore size; Millipore) by wet electroblotting (200 mA for 90 min). Membranes, incubated with a HRP-conjugated secondary antibody (K1223, APExBIO; 1:5000), were developed with ECL (WBKLS0100, Millipore). #Weight marker (molecular weight in KD): Thermo ScientificTM/PageRulerTM Prestained Protein Ladder, 10 to 180 kDa; catalogue number: 26616.

FIGURE5D

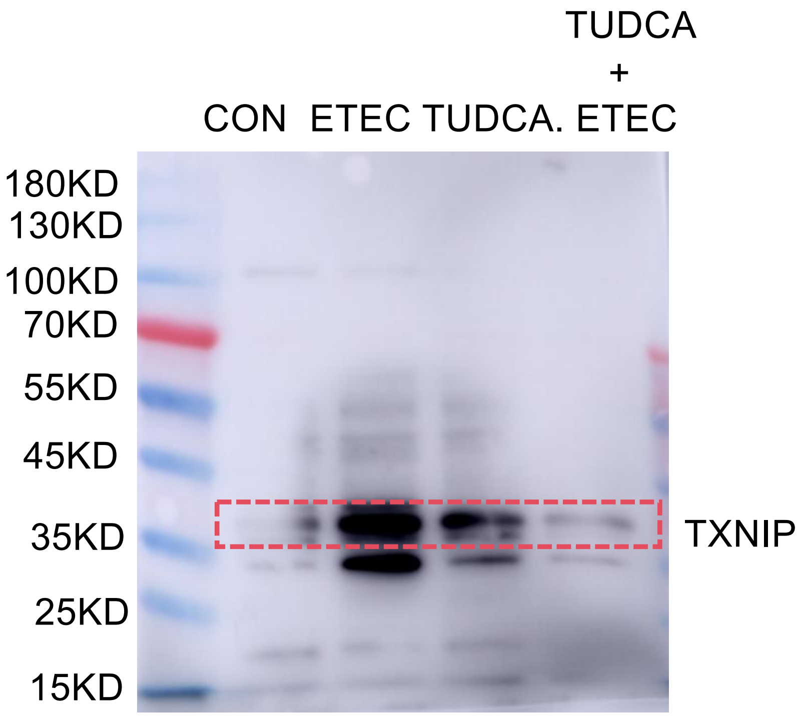

Figure 5D. Western blot membrane of TXNIP (~37 kDa) protein detected with anti-TXNIP (AF8277, Beyotime; 1:1000) antibody. Gel-separated proteins were transferred to PVDF (0.45 µm pore size; Millipore) by wet electroblotting (200 mA for 90 min). Membranes, incubated with a HRP-conjugated secondary antibody (K1223, APExBIO; 1:5000), were developed with ECL (WBKLS0100, Millipore). #Weight marker (molecular weight in KD): Thermo ScientificTM/PageRulerTM Prestained Protein Ladder, 10 to 180 kDa; catalogue number: 26616.

FIGURE5D

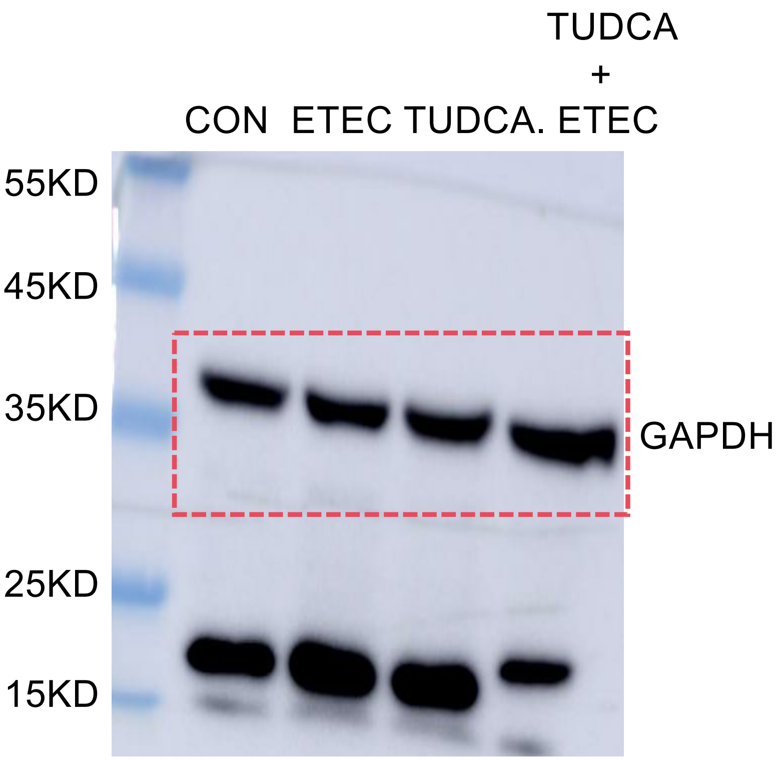

Figure 5D. Western blot membrane of GAPDH (~37 kDa) protein detected with anti-GAPDH (AF7021, Affinity Bioscience; 1:3000) antibody. Gel-separated proteins were transferred to PVDF (0.45 µm pore size; Millipore) by wet electroblotting (200 mA for 90 min). Membranes, incubated with a HRP-conjugated secondary antibody (K1223, APExBIO; 1:5000), were developed with ECL (WBKLS0100, Millipore). #Weight marker (molecular weight in KD): Thermo ScientificTM/PageRulerTM Prestained Protein Ladder, 10 to 180 kDa; catalogue number: 26616.

FIGURE5F

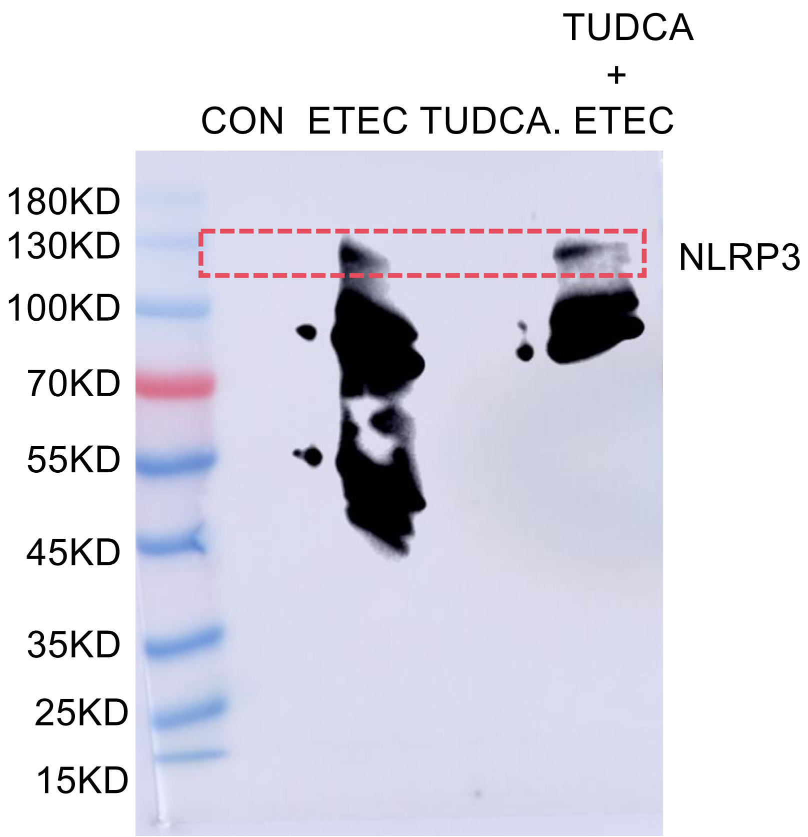

Figure 5F. Western blot membrane of NLRP3 (~110 kDa) protein detected with anti-NLRP3 (A5620, Abclonal; 1:1000) antibody. Gel-separated proteins were transferred to PVDF (0.45 µm pore size; Millipore) by wet electroblotting (200 mA for 90 min). Membranes, incubated with a HRP-conjugated secondary antibody (K1223, APExBIO; 1:5000), were developed with ECL (WBKLS0100, Millipore). #Weight marker (molecular weight in KD): Thermo ScientificTM/PageRulerTM Prestained Protein Ladder, 10 to 180 kDa; catalogue number: 26616.

FIGURE5F

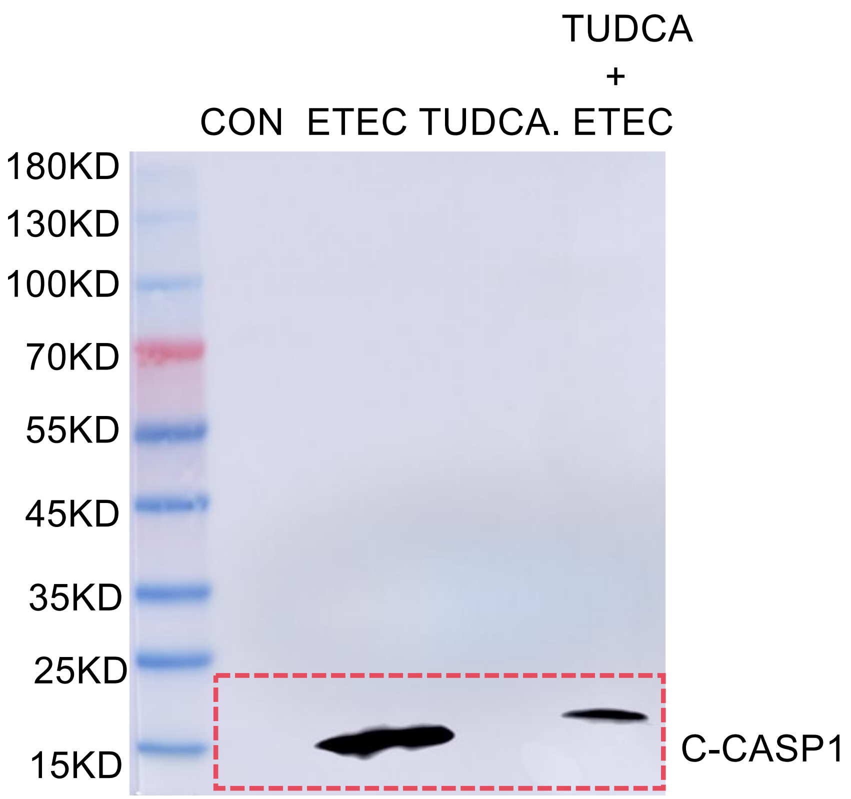

Figure 5F. Western blot membrane of cleaved-Caspase-1 (C-CASP1) (~19 kDa) protein detected with anti-Caspase-1 (AF5418, Affinity Bioscience; 1:1000) antibody. Gel-separated proteins were transferred to PVDF (0.45 µm pore size; Millipore) by wet electroblotting (200 mA for 90 min). Membranes, incubated with a HRP-conjugated secondary antibody (K1223, APExBIO; 1:5000), were developed with ECL (WBKLS0100, Millipore). #Weight marker (molecular weight in KD): Thermo ScientificTM/PageRulerTM Prestained Protein Ladder, 10 to 180 kDa; catalogue number: 26616.

FIGURE5F

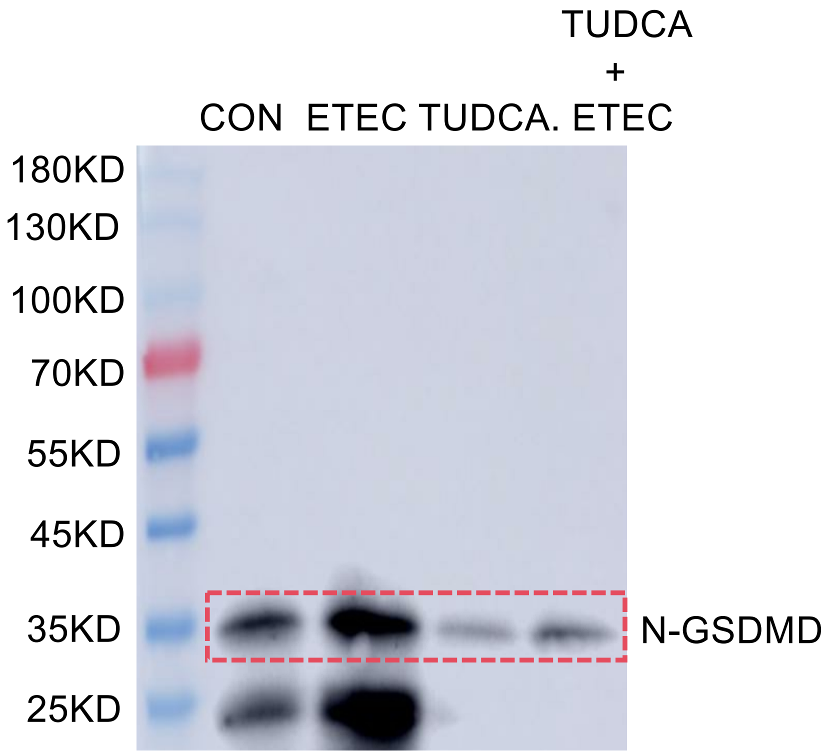

Figure 5F. Western blot membrane of N terminal GSDMD (N-GSDMD) (~35 kDa) protein detected with anti-GSDMD (AF4012, Affinity Bioscience ; 1:1000) antibody. Gel-separated proteins were transferred to PVDF (0.45 µm pore size; Millipore) by wet electroblotting (200 mA for 90 min). Membranes, incubated with a HRP-conjugated secondary antibody (K1223, APExBIO; 1:5000), were developed with ECL (WBKLS0100, Millipore). #Weight marker (molecular weight in KD): Thermo ScientificTM/PageRulerTM Prestained Protein Ladder, 10 to 180 kDa; catalogue number: 26616.

FIGURE5F

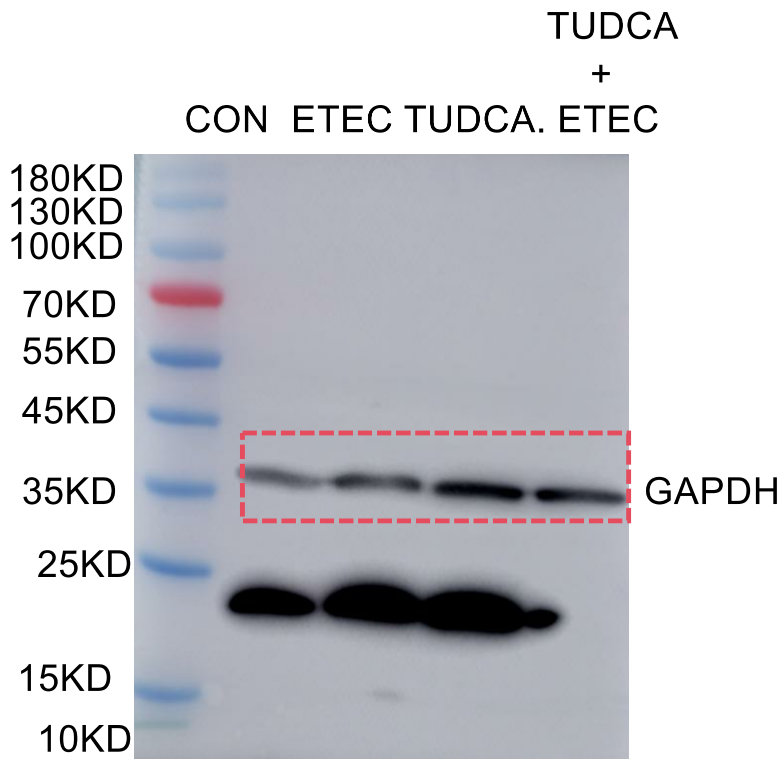

Figure 5F. Western blot membrane of GAPDH (~37 kDa) protein detected with anti-GAPDH (AF7021, Affinity Bioscience; 1:3000) antibody. Gel-separated proteins were transferred to PVDF (0.45 µm pore size; Millipore) by wet electroblotting (200 mA for 90 min). Membranes, incubated with a HRP-conjugated secondary antibody (K1223, APExBIO; 1:5000), were developed with ECL (WBKLS0100, Millipore). #Weight marker (molecular weight in KD): Thermo ScientificTM/PageRulerTM Prestained Protein Ladder, 10 to 180 kDa; catalogue number: 26616.

FIGURE7A

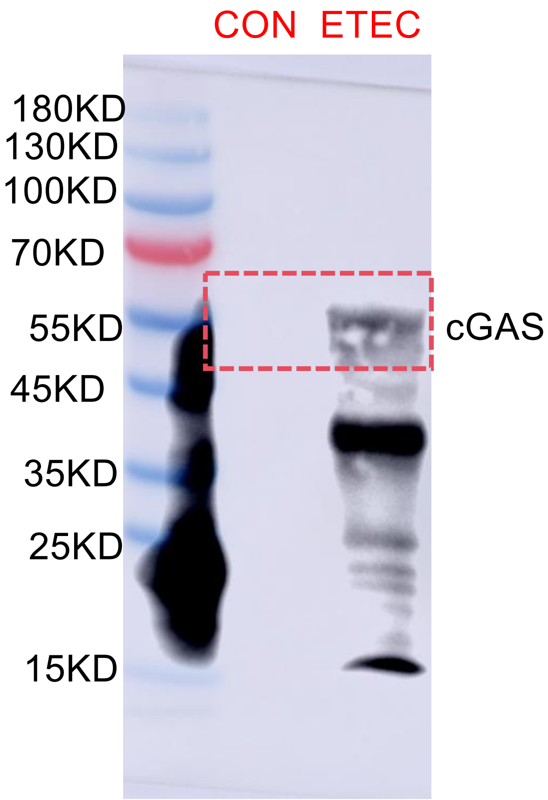

Figure 7A. Western blot membrane of cGAS (~62 kDa) protein detected with anti-cGAS (A8335, Abclonal; 1:1000) antibody. Gel-separated proteins were transferred to PVDF (0.45 µm pore size; Millipore) by wet electroblotting (200 mA for 90 min). Membranes, incubated with a HRP-conjugated secondary antibody (K1223, APExBIO; 1:5000), were developed with ECL (WBKLS0100, Millipore). #Weight marker (molecular weight in KD): Thermo ScientificTM/PageRulerTM Prestained Protein Ladder, 10 to 180 kDa; catalogue number: 26616.

FIGURE7A

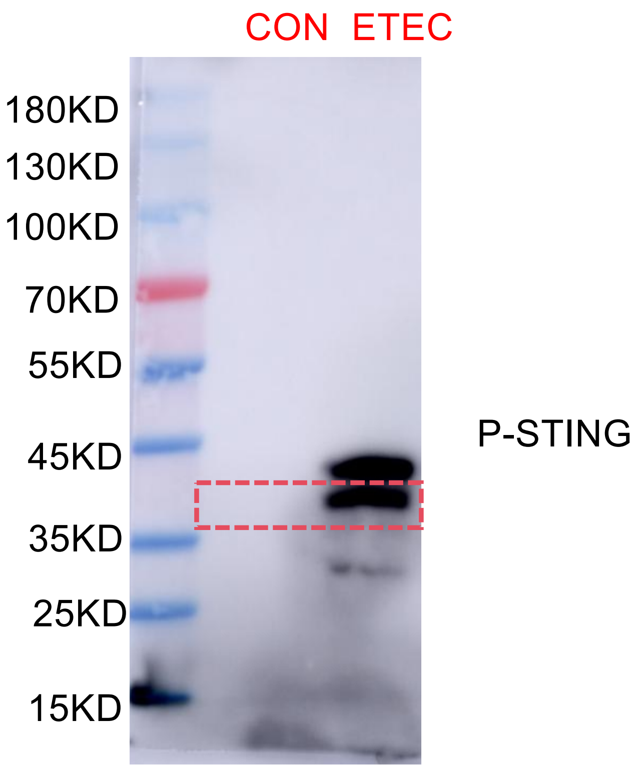

Figure 7A. Western blot membrane of phosphorylated STING (P-STING) (~42 kDa) protein detected with anti-P-STING (AP1369, Abclonal; 1:1000) antibody. Gel-separated proteins were transferred to PVDF (0.45 µm pore size; Millipore) by wet electroblotting (200 mA for 90 min). Membranes, incubated with a HRP-conjugated secondary antibody (K1223, APExBIO; 1:5000), were developed with ECL (WBKLS0100, Millipore). #Weight marker (molecular weight in KD): Thermo ScientificTM/PageRulerTM Prestained Protein Ladder, 10 to 180 kDa; catalogue number: 26616.

FIGURE7A

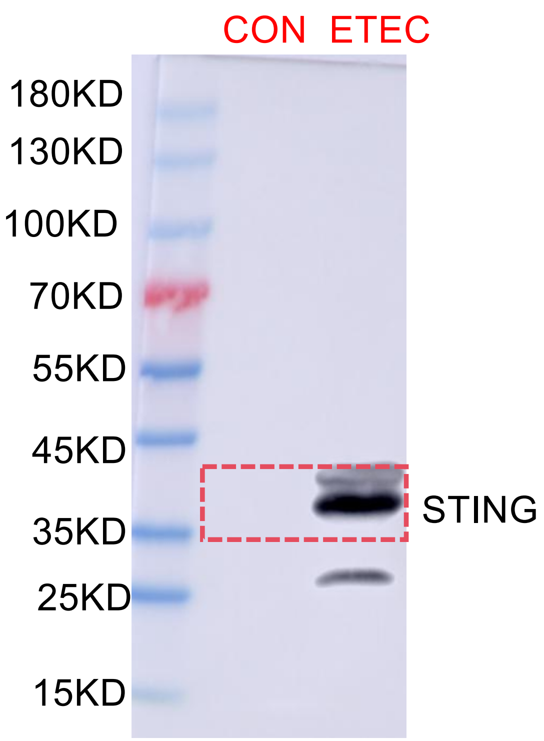

Figure 7A. Western blot membrane of STING (~42 kDa) protein detected with anti-STING (AP1369, Abclonal; 1:1000) antibody. Gel-separated proteins were transferred to PVDF (0.45 µm pore size; Millipore) by wet electroblotting (200 mA for 90 min). Membranes, incubated with a HRP-conjugated secondary antibody (K1223, APExBIO; 1:5000), were developed with ECL (WBKLS0100, Millipore). #Weight marker (molecular weight in KD): Thermo ScientificTM/PageRulerTM Prestained Protein Ladder, 10 to 180 kDa; catalogue number: 26616.

FIGURE7A

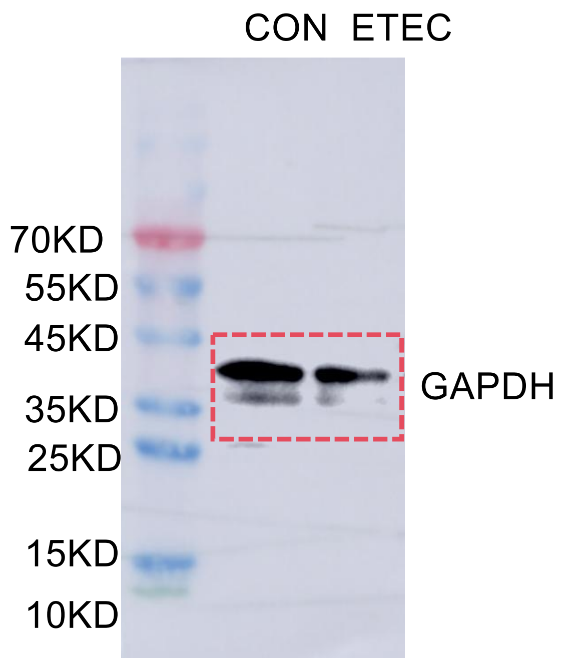

Figure 7A. Western blot membrane of GAPDH (~37 kDa) protein detected with anti-GAPDH (AF7021, Affinity Bioscience; 1:3000) antibody. Gel-separated proteins were transferred to PVDF (0.45 µm pore size; Millipore) by wet electroblotting (200 mA for 90 min). Membranes, incubated with a HRP-conjugated secondary antibody (K1223, APExBIO; 1:5000), were developed with ECL (WBKLS0100, Millipore). #Weight marker (molecular weight in KD): Thermo ScientificTM/PageRulerTM Prestained Protein Ladder, 10 to 180 kDa; catalogue number: 26616.

FIGURE7B

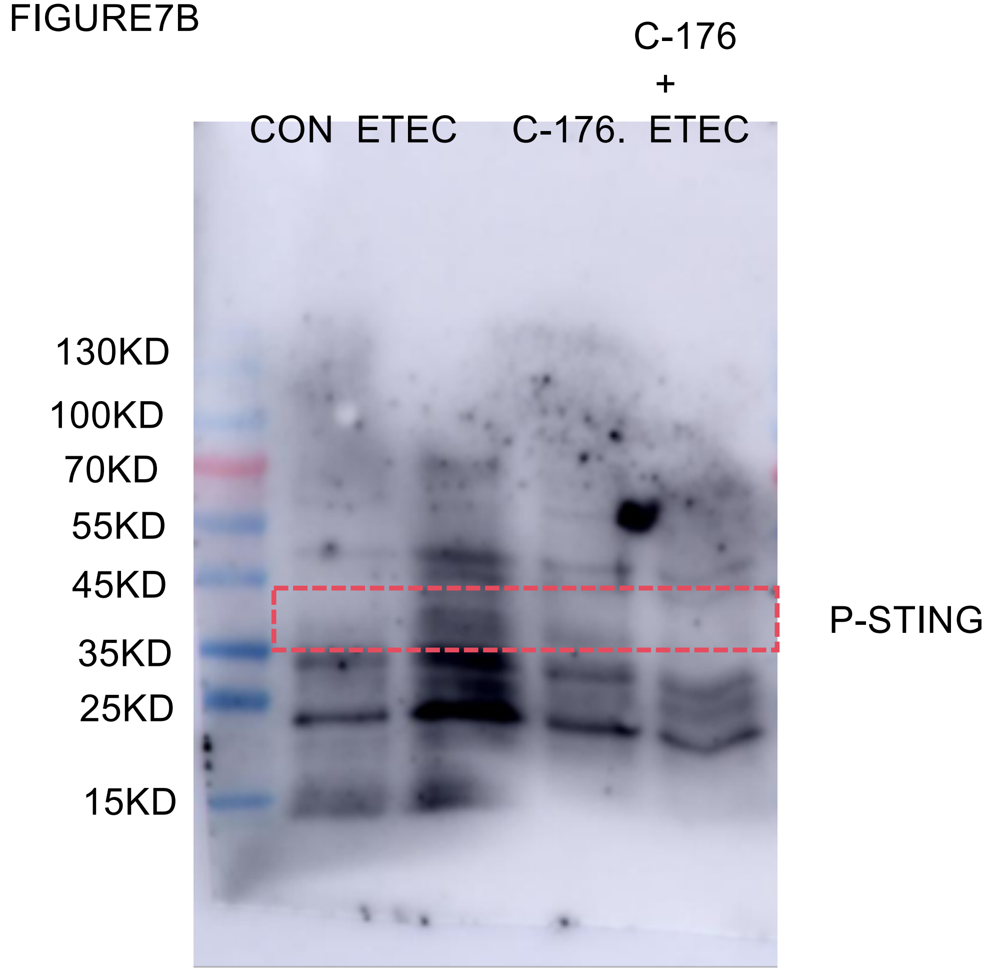

Figure 7B. Western blot membrane of phosphorylated STING (P-STING) (~42 kDa) protein detected with anti-P-STING (AP1369, Abclonal; 1:1000) antibody. Gel-separated proteins were transferred to PVDF (0.45 µm pore size; Millipore) by wet electroblotting (200 mA for 90 min). Membranes, incubated with a HRP-conjugated secondary antibody (K1223, APExBIO; 1:5000), were developed with ECL (WBKLS0100, Millipore). #Weight marker (molecular weight in KD): Thermo ScientificTM/PageRulerTM Prestained Protein Ladder, 10 to 180 kDa; catalogue number: 26616.

FIGURE7B

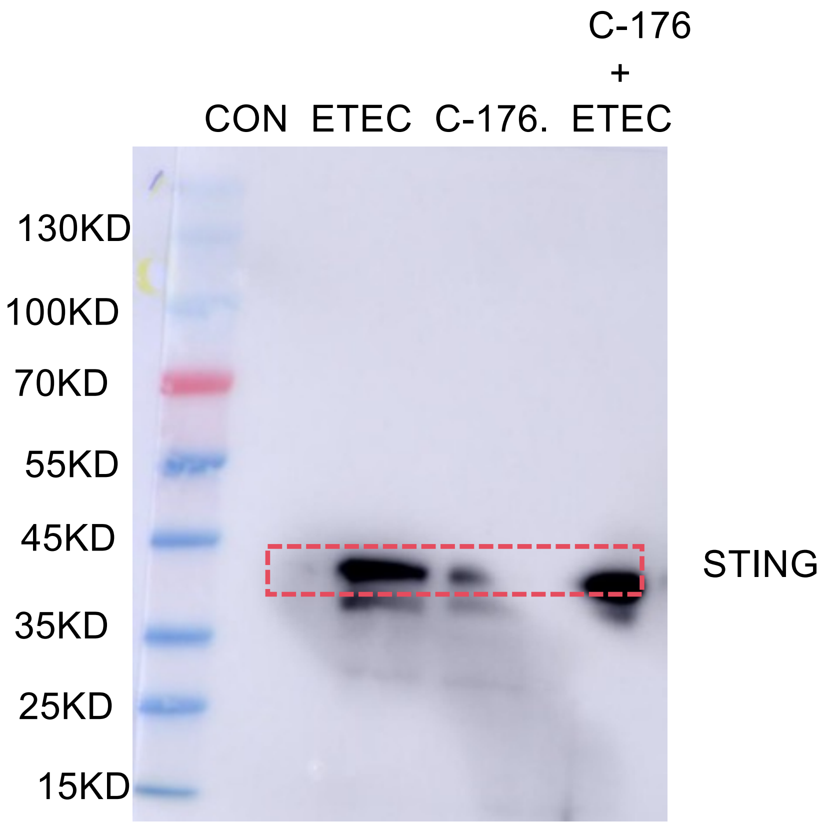

Figure 7B. Western blot membrane of STING (~42 kDa) protein detected with anti-STING (AP1369, Abclonal; 1:1000) antibody. Gel-separated proteins were transferred to PVDF (0.45 μm pore size; Millipore) by wet electroblotting (200 mA for 90 min). Membranes, incubated with a HRP-conjugated secondary antibody (K1223, APExBIO; 1:5000), were developed with ECL (WBKLS0100, Millipore). #Weight marker (molecular weight in KD): Thermo ScientificTM/PageRulerTM Prestained Protein Ladder, 10 to 180 kDa; catalogue number: 26616.

FIGURE7B

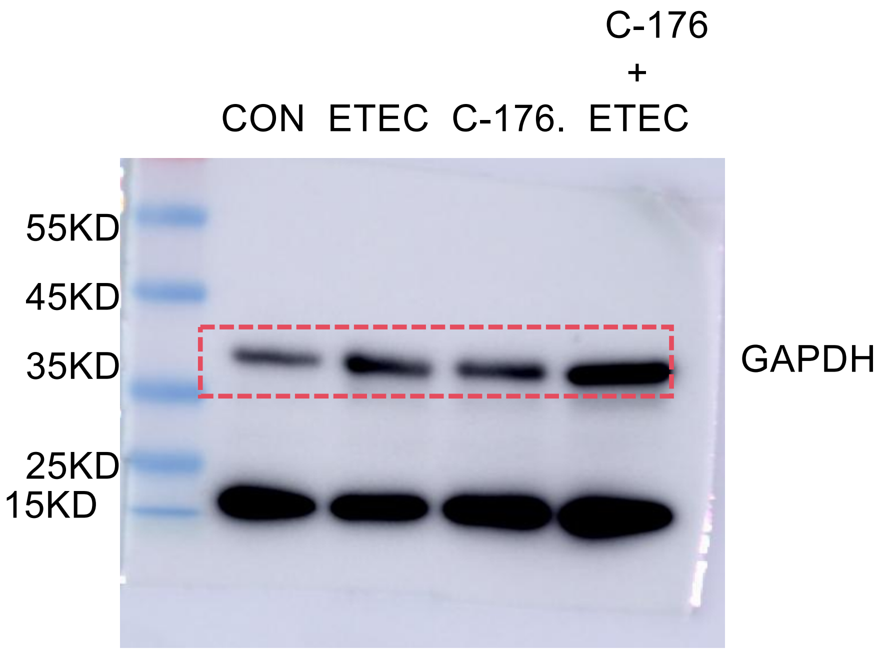

Figure 7B. Western blot membrane of GAPDH (~37 kDa) protein detected with anti-GAPDH (AF7021, Affinity Bioscience; 1:3000) antibody. Gel-separated proteins were transferred to PVDF (0.45 μm pore size; Millipore) by wet electroblotting (200 mA for 90 min). Membranes, incubated with a HRP-conjugated secondary antibody (K1223, APExBIO; 1:5000), were developed with ECL (WBKLS0100, Millipore). #Weight marker (molecular weight in KD): Thermo ScientificTM/PageRulerTM Prestained Protein Ladder, 10 to 180 kDa; catalogue number: 26616.

FIGURE7D

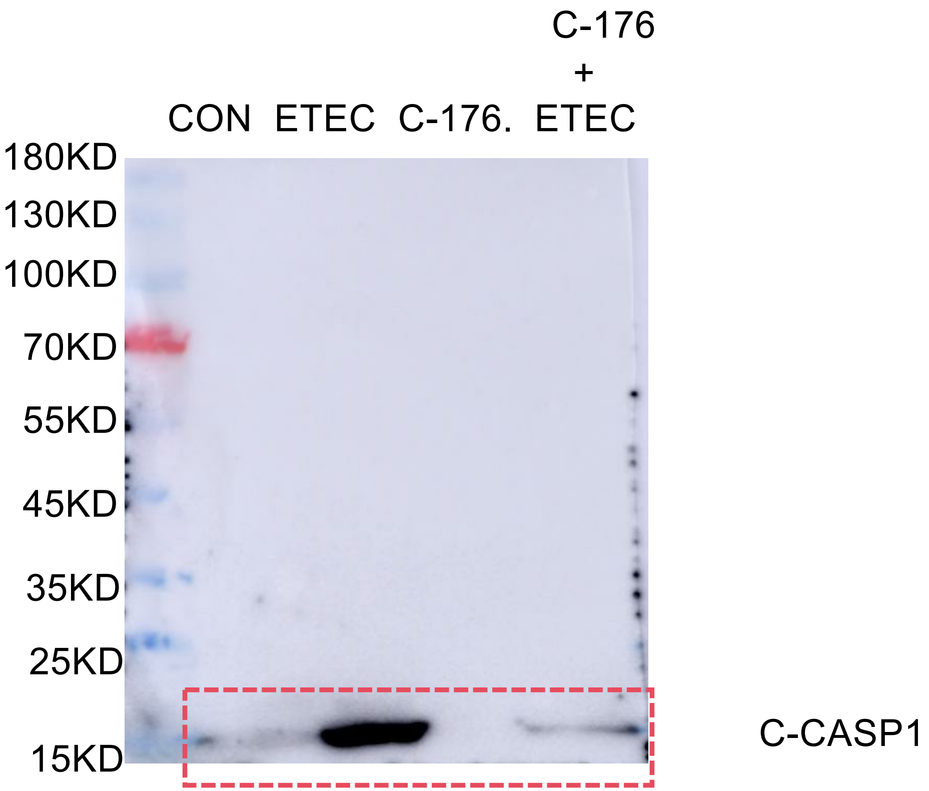

Figure 7D. Western blot membrane of cleaved-Caspase-1 (C-CASP1) (~19 kDa) protein detected with anti-Caspase-1 (AF5418, Affinity Bioscience; 1:1000) antibody. Gel-separated proteins were transferred to PVDF (0.45 μm pore size; Millipore) by wet electroblotting (200 mA for 90 min). Membranes, incubated with a HRP-conjugated secondary antibody (K1223, APExBIO; 1:5000), were developed with ECL (WBKLS0100, Millipore). #Weight marker (molecular weight in KD): Thermo ScientificTM/PageRulerTM Prestained Protein Ladder, 10 to 180 kDa; catalogue number: 26616.

FIGURE7D

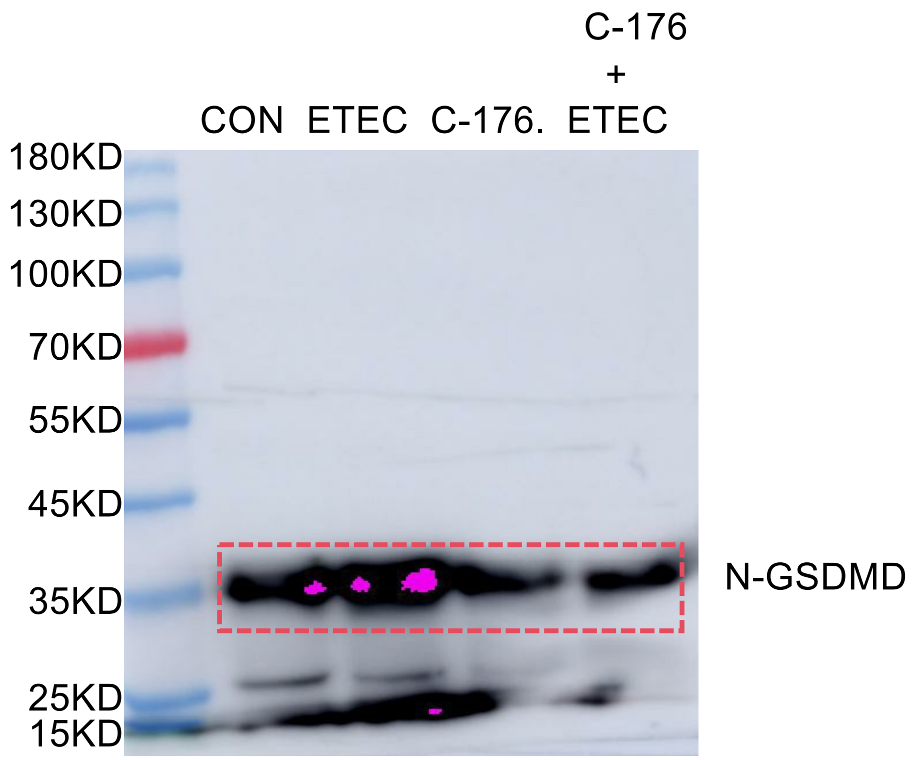

Figure 7D. Western blot membrane of N terminal GSDMD (N-GSDMD) (~35 kDa) protein detected with anti-GSDMD (AF4012, Affinity Bioscience ; 1:1000) antibody. Gel-separated proteins were transferred to PVDF (0.45 µm pore size; Millipore) by wet electroblotting (200 mA for 90 min). Membranes, incubated with a HRP-conjugated secondary antibody (K1223, APExBIO; 1:5000), were developed with ECL (WBKLS0100, Millipore). #Weight marker (molecular weight in KD): Thermo ScientificTM/PageRulerTM Prestained Protein Ladder, 10 to 180 kDa; catalogue number: 26616.

FIGURE7D

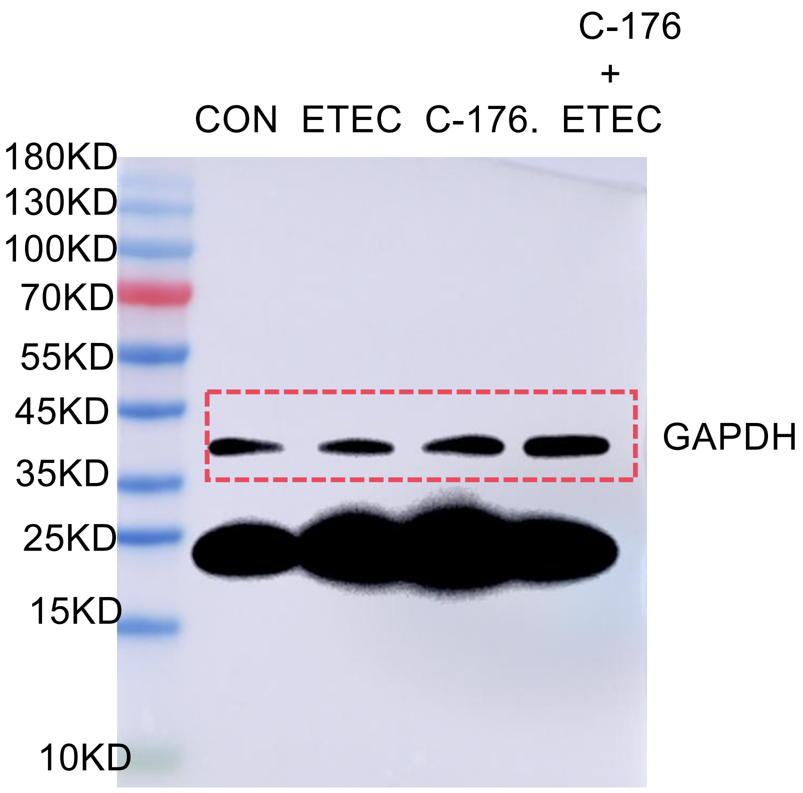

Figure 7D. Western blot membrane of GAPDH (~37 kDa) protein detected with anti-GAPDH (AF7021, Affinity Bioscience; 1:3000) antibody. Gel-separated proteins were transferred to PVDF (0.45 µm pore size; Millipore) by wet electroblotting (200 mA for 90 min). Membranes, incubated with a HRP-conjugated secondary antibody (K1223, APExBIO; 1:5000), were developed with ECL (WBKLS0100, Millipore). #Weight marker (molecular weight in KD): Thermo ScientificTM/PageRulerTM Prestained Protein Ladder, 10 to 180 kDa; catalogue number: 26616.

FIGURE7E

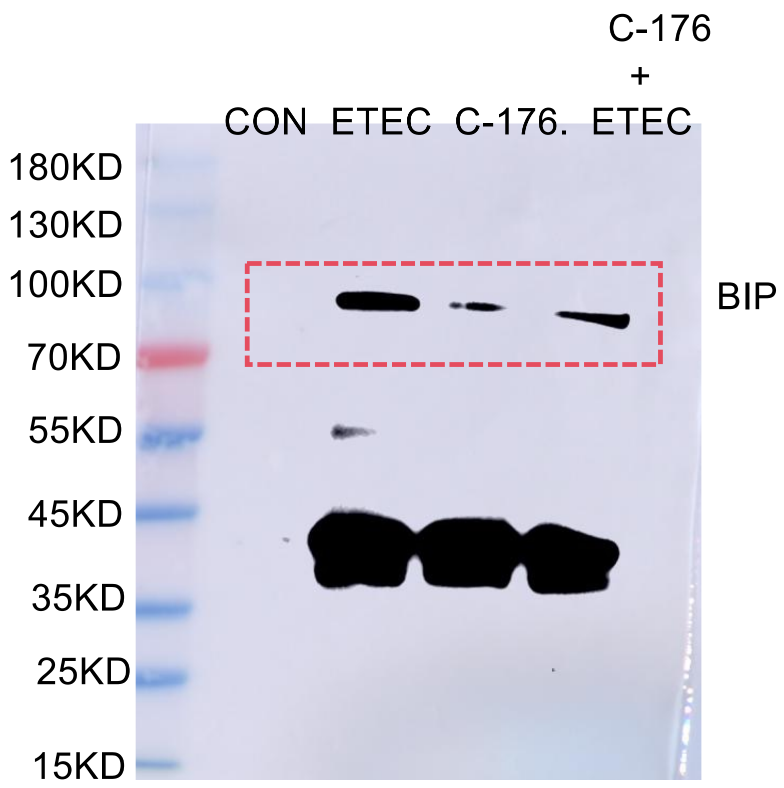

Figure 7E. Western blot membrane of BIP (~78 kDa) protein detected with anti-BIP (AF5366, Affinity Bioscience; 1:1000) antibody. Gel-separated proteins were transferred to PVDF (0.45 µm pore size; Millipore) by wet electroblotting (200 mA for 90 min). Membranes, incubated with a HRP-conjugated secondary antibody (K1223, APExBIO; 1:5000), were developed with ECL (WBKLS0100, Millipore). #Weight marker (molecular weight in KD): Thermo ScientificTM/PageRulerTM Prestained Protein Ladder, 10 to 180 kDa; catalogue number: 26616.

FIGURE7E

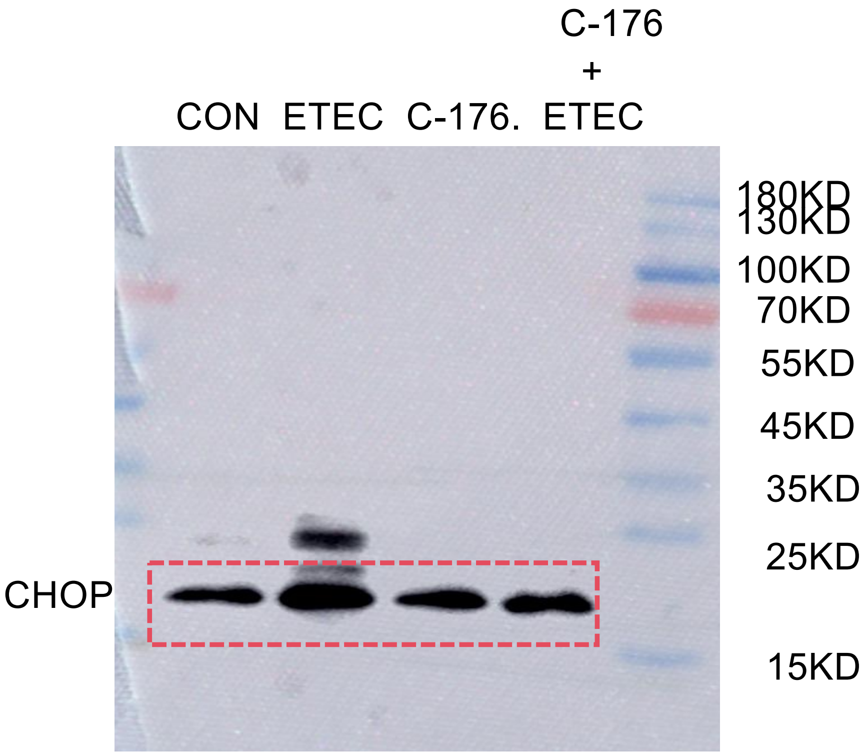

Figure 7E. Western blot membrane of CHOP (~19 kDa) protein detected with anti-CHOP (AF6277, Affinity Bioscience; 1:1000) antibody. Gel-separated proteins were transferred to PVDF (0.45 μm pore size; Millipore) by wet electroblotting (200 mA for 90 min). Membranes, incubated with a HRP-conjugated secondary antibody (K1223, APExBIO; 1:5000), were developed with ECL (WBKLS0100, Millipore). #Weight marker (molecular weight in KD): Thermo ScientificTM/PageRulerTM Prestained Protein Ladder, 10 to 180 kDa; catalogue number: 26616.

FIGURE7E

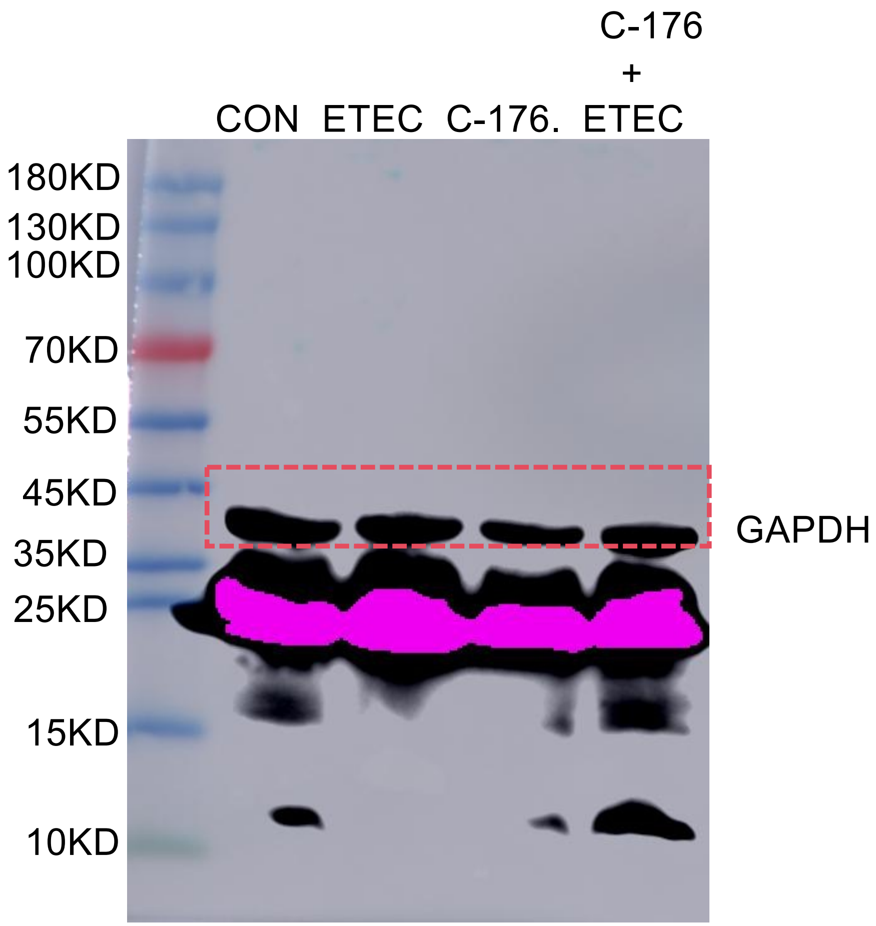

Figure 7E. Western blot membrane of GAPDH (~37 kDa) protein detected with anti-GAPDH (AF7021, Affinity Bioscience; 1:3000) antibody. Gel-separated proteins were transferred to PVDF (0.45 μm pore size; Millipore) by wet electroblotting (200 mA for 90 min). Membranes, incubated with a HRP-conjugated secondary antibody (K1223, APExBIO; 1:5000), were developed with ECL (WBKLS0100, Millipore). #Weight marker (molecular weight in KD): Thermo ScientificTM/PageRulerTM Prestained Protein Ladder, 10 to 180 kDa; catalogue number: 26616.

FIGURE7F

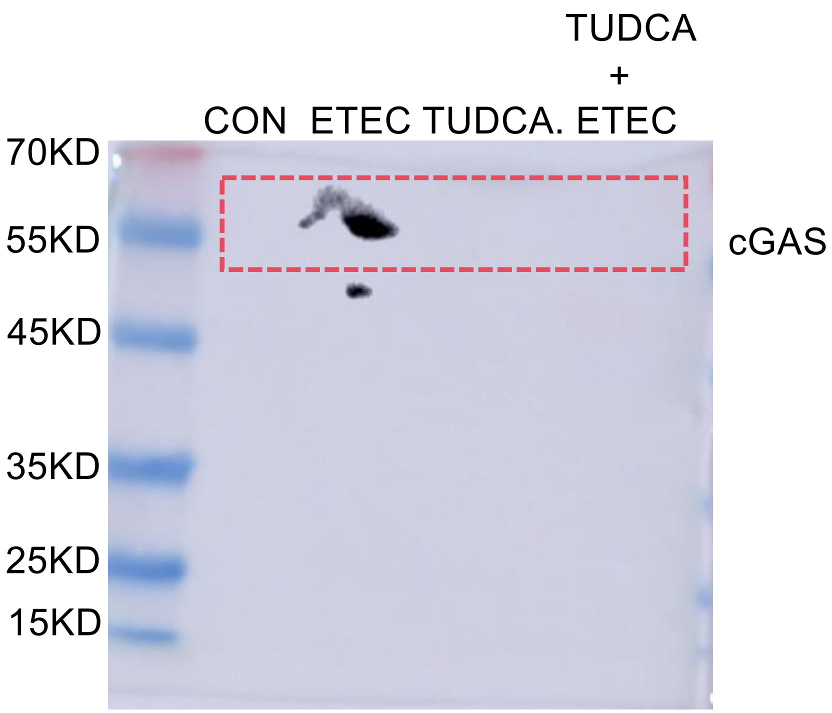

Figure 7F. Western blot membrane of cGAS (~62 kDa) protein detected with anti-cGAS (A8335, Abclonal; 1:1000) antibody. Gel-separated proteins were transferred to PVDF (0.45 µm pore size; Millipore) by wet electroblotting (200 mA for 90 min). Membranes, incubated with a HRP-conjugated secondary antibody (K1223, APExBIO; 1:5000), were developed with ECL (WBKLS0100, Millipore). #Weight marker (molecular weight in KD): Thermo ScientificTM/PageRulerTM Prestained Protein Ladder, 10 to 180 kDa; catalogue number: 26616.

FIGURE7F

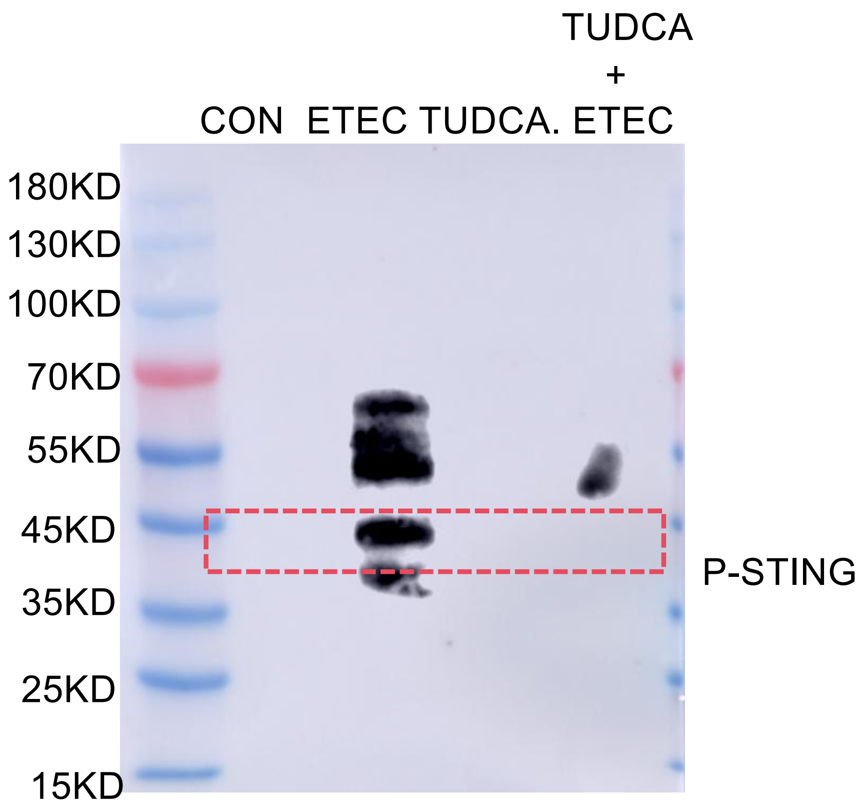

Figure 7F. Western blot membrane of phosphorylated STING (P-STING) (~42 kDa) protein detected with anti-P-STING (AP1369, Abclonal; 1:1000) antibody. Gel-separated proteins were transferred to PVDF (0.45 µm pore size; Millipore) by wet electroblotting (200 mA for 90 min). Membranes, incubated with a HRP-conjugated secondary antibody (K1223, APExBIO; 1:5000), were developed with ECL (WBKLS0100, Millipore). #Weight marker (molecular weight in KD): Thermo ScientificTM/PageRulerTM Prestained Protein Ladder, 10 to 180 kDa; catalogue number: 26616.

FIGURE7F

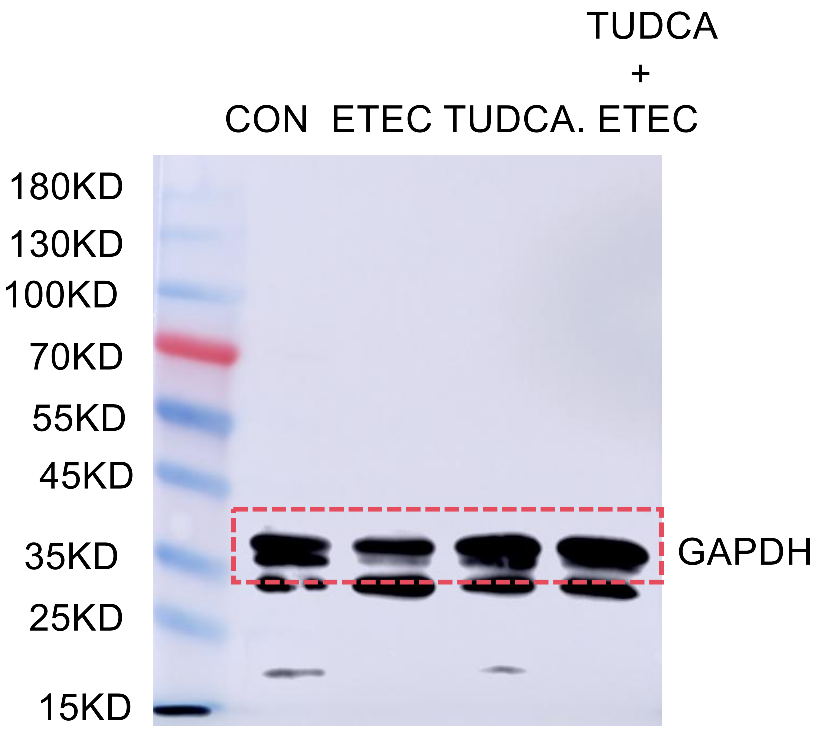

Figure 7F. Western blot membrane of GAPDH (~37 kDa) protein detected with anti-GAPDH (AF7021, Affinity Bioscience; 1:3000) antibody. Gel-separated proteins were transferred to PVDF (0.45 µm pore size; Millipore) by wet electroblotting (200 mA for 90 min). Membranes, incubated with a HRP-conjugated secondary antibody (K1223, APExBIO; 1:5000), were developed with ECL (WBKLS0100, Millipore). #Weight marker (molecular weight in KD): Thermo ScientificTM/PageRulerTM Prestained Protein Ladder, 10 to 180 kDa; catalogue number: 26616.

FIGURE S1C

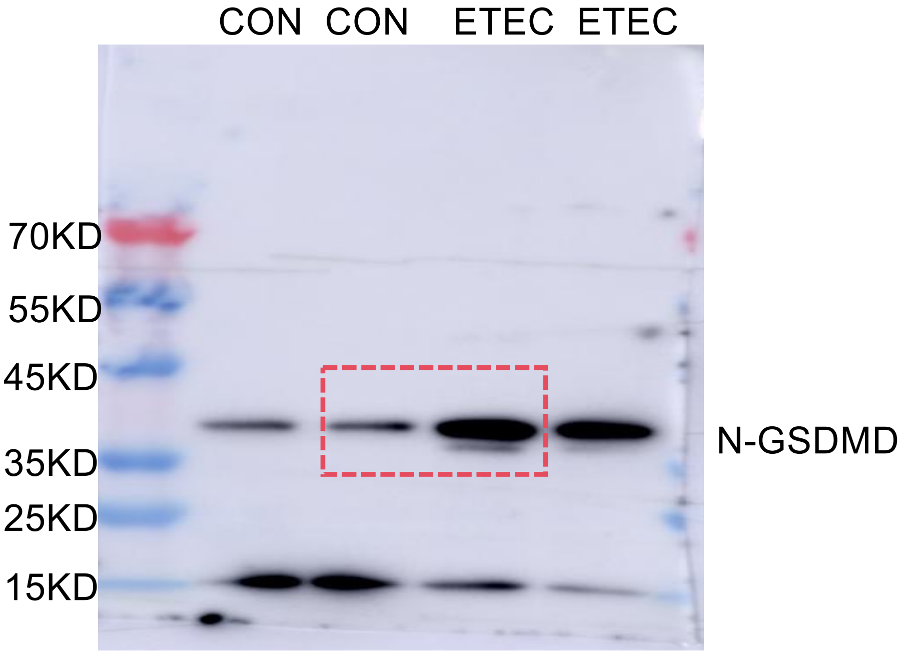

Figure S1C. Western blot membrane of N terminal GSDMD (N-GSDMD) (~35 kDa) protein detected with anti-GSDMD (AF4012, Affinity Bioscience ; 1:1000) antibody. Gel-separated proteins were transferred to PVDF (0.45 µm pore size; Millipore) by wet electroblotting (200 mA for 90 min). Membranes, incubated with a HRP-conjugated secondary antibody (K1223, APExBIO; 1:5000), were developed with ECL (WBKLS0100, Millipore). #Weight marker (molecular weight in KD): Thermo ScientificTM/PageRulerTM Prestained Protein Ladder, 10 to 180 kDa; catalogue number: 26616.

FIGURE S1C

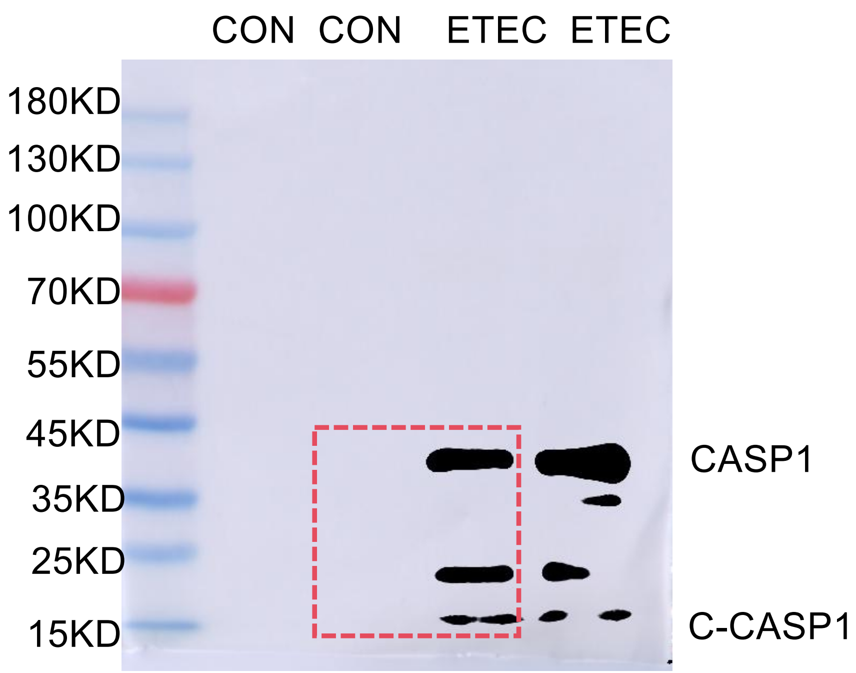

Figure S1C. Western blot membrane of Caspase-1 (CASP1) (~37 kDa) and cleaved-Caspase-1 (C-CASP1) (~19 kDa) protein detected with anti-Caspase-1 (AF5418, Affinity Bioscience; 1:1000) antibody. Gel-separated proteins were transferred to PVDF (0.45 µm pore size; Millipore) by wet electroblotting (200 mA for 90 min). Membranes, incubated with a HRP-conjugated secondary antibody (K1223, APExBIO; 1:5000), were developed with ECL (WBKLS0100, Millipore). #Weight marker (molecular weight in KD): Thermo ScientificTM/PageRulerTM Prestained Protein Ladder, 10 to 180 kDa; catalogue number: 26616.

FIGURE S1C

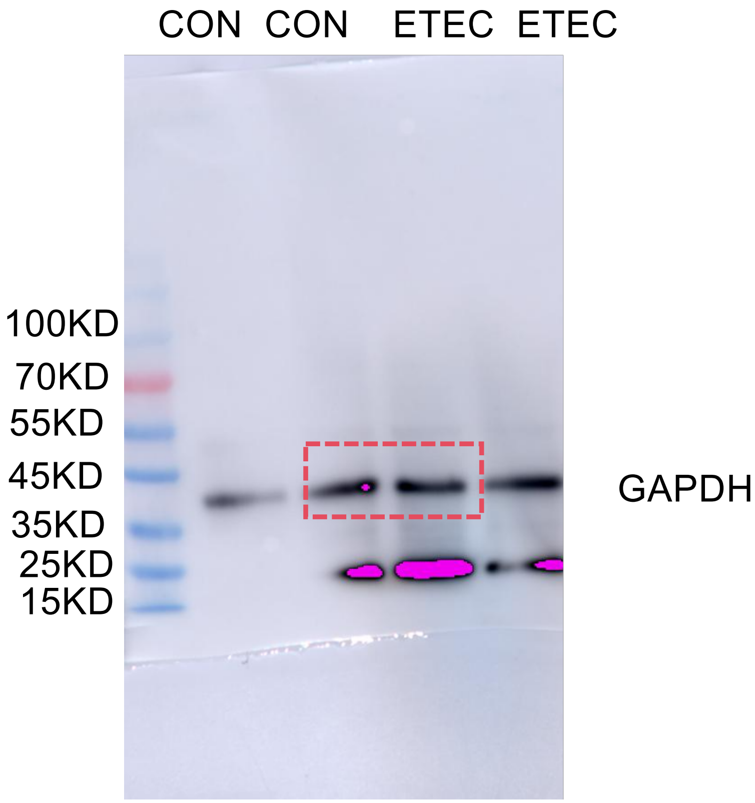

Figure S1C. Western blot membrane of GAPDH (~37 kDa) protein detected with anti-GAPDH (AF7021, Affinity Bioscience; 1:3000) antibody. Gel-separated proteins were transferred to PVDF (0.45 µm pore size; Millipore) by wet electroblotting (200 mA for 90 min). Membranes, incubated with a HRP-conjugated secondary antibody (K1223, APExBIO; 1:5000), were developed with ECL (WBKLS0100, Millipore). #Weight marker (molecular weight in KD): Thermo ScientificTM/PageRulerTM Prestained Protein Ladder, 10 to 180 kDa; catalogue number: 26616.
